# Quantitative electroencephalogram utility in predicting conversion of mild cognitive impairment to dementia with Lewy bodies^☆^

**DOI:** 10.1016/j.neurobiolaging.2014.07.009

**Published:** 2015-01

**Authors:** Laura Bonanni, Bernardo Perfetti, Stefania Bifolchetti, John-Paul Taylor, Raffaella Franciotti, Lucilla Parnetti, Astrid Thomas, Marco Onofrj

**Affiliations:** a Department of Neuroscience and Imaging, G. d'Annunzio University, Chieti, Italy; b Aging Research Centre, Ce.S.I., G. d'Annunzio University Foundation, Chieti, Italy; c Institute for Ageing and Health, Newcastle University, Campus for Ageing and Vitality, Newcastle upon Tyne, UK; d Institute for Advanced Biomedical Technologies (ITAB), G. d'Annunzio University Foundation, Chieti, Italy; e Center for Memory Disturbances and Alzheimer's Center, Section of Neurology, University of Perugia, Perugia, Italy

**Keywords:** Mild cognitive impairment, Dementia with Lewy bodies, Quantitative EEG

## Abstract

Mild cognitive impairment (MCI) as a precursor of dementia with Lewy bodies (DLB) is the focus of recent research, trying to explore the early mechanisms and possible biomarkers of DLB. Quantitative electroencephalogram (QEEG) methods are able to differentiate early DLB from Alzheimer's disease (AD). The aim of the present study was to assess whether QEEG abnormalities, characterized by dominant frequency <8 Hz and dominant frequency variability >1.5 Hz, typical of early DLB, are already present at the stage of MCI and to evaluate whether EEG abnormalities can predict the development of DLB. Forty-seven MCI subjects were followed for 3 years. EEG recordings were obtained at admission and at the end of the study. At the end of follow-up, 20 subjects had developed probable DLB (MCI-DLB), 14 had probable AD (MCI-AD), 8 did not convert to dementia, 5 developed a non-AD/DLB dementia. One hundred percent of MCI-DLB showed EEG abnormalities at admission. Ninety three percent of MCI-AD maintained a normal EEG throughout the study. QEEG may represent a powerful tool to predict the progression from MCI to DLB with a sensitivity and specificity close to 100%.

## Highlights

• We studied in mild cognitive impairment (MCI) subjects the predictive value of electroencephalogram (EEG) alterations for the development of dementia with Lewy bodies (DLB).
• One hundred percent of MCI subjects converted to DLB already had EEG alterations typical of DLB (dominant frequency <8 Hz and dominant frequency variability ≥1.5 Hz) at admission to the study.
• Quantitative EEG may represent a powerful tool to predict the progression from MCI to DLB.

## 1 Introduction

The early identification of dementia is becoming increasingly important, as it is likely that it is during this time period, before the manifestation of significant pathophysiological change that disease modifying treatments will have their biggest impact. Dementia is generally preceded by an early preclinical phase, which progresses to mild cognitive impairment (MCI) and finally to dementia (Albert et al., 2011). The observations and evaluation of MCI patients through neuropsychological tools designed to assess different cognitive domains (such as memory, executive functions, and visuospatial skills) commonly applied to patients with dementia, allow the definition of 2 main MCI subtypes: amnestic MCI (aMCI), which presents with dominant memory function impairment; and nonamnestic MCI (naMCI), which presents with prominent impairment of cognitive domains other than memory, and includes attention, language, executive functions, visuospatial skills.

In both MCI subtypes, the cognitive impairment can be restricted only to a specific domain (e.g., memory and attention), defining the so-called single-domain MCI, or can present as a combination of dysfunctions in more than 1 cognitive domain, defining the so-called multiple domain MCI (Winblad et al., 2004).

It has been proposed that the different subtypes of MCI are associated with progression to different dementia types.

Specifically, patients with amnestic MCI are considered more likely to progress to Alzheimer's disease (AD) (Petersen et al., 2001), whereas patients with naMCI are more likely to progress to a non-AD dementia, including, for example, dementia with Lewy bodies (DLB) (Boeve, 2012).

Currently, great efforts are being put toward the early identification of preclinical, biological, clinical, laboratory markers which are able to predict the conversion of MCI to AD (Sperling et al., 2011).

An example of a successful biomarker of conversion from MCI to AD is the analysis of proteins present in the cerebrospinal fluid (CSF) and in particular total tau (tau), phosphorylated tau (P-tau), and the 42-amino-acid isoform of amyloid-β_1–42_ (Aβ_42_) (Parnetti et al., 2012). Similarly, structural and functional neuroimaging studies have provided biomarkers for the conversion of MCI to AD (Tosun et al., 2013).

A further promising approach to assess the conversion of MCI subjects to AD subjects or to study the progression of AD from mild to more pronounced stages of dementia is the recording of resting state eyes-closed electroencephalographic (EEG) rhythms.

Cortical sources of resting state EEG rhythms in mild AD patients are sensitive to the disease progression at the early stage over 1 year (Babiloni et al., 2013). In particular, follow-up EEG recordings (Babiloni et al., 2013) have demonstrated that alterations of EEG cortical rhythms characterized either by increased power of widespread delta sources and decreased power of alpha and posterior beta (13–20 Hz) sources in mild AD patients or by decreased power of posterior alpha sources in amnestic MCI subjects correlate with cognitive decline (Babiloni et al., 2013, Babiloni et al., 2014).

However, although MCI as a prodromal condition for AD is a well studied and characterized condition, (Petersen et al., 2001), MCI associated with Lewy body disease (including DLB and PDD), which represents the second most common form of neurodegenerative dementia and associated with highly distressing behavioral symptoms (McKeith et al., 2005) appears to be less typified in literature (Auning et al., 2011, Burn and Barker, 2013, Litvan et al., 2011).

EEG has extensively been studied as a possible tool to assess the presence of dementia (Breslau et al., 1989, Briel et al., 1999, Giaquinto and Nolfe, 1986), and in Consensus criteria for the diagnosis of DLB, EEG abnormalities are described among the supportive features for the diagnosis of DLB (McKeith, 2005).

Several studies suggested that EEG analyzed with quantitative methods is able to differentiate with high specificity and sensitivity, DLB from AD from the very early stages of disease (Andersson et al., 2008, Bonanni et al., 2008, Franciotti et al., 2006, Walker et al., 2000), and these alterations of electrocortical arousal are highly correlated with the presence of fluctuating cognition (Andersson et al., 2008, Bonanni et al., 2008, Franciotti et al., 2006, Walker et al., 2000), a core symptom for the diagnosis of DLB, which among the various clinical features proposed for DLB diagnosis, has been demonstrated to be the most specific (Tiraboschi et al., 2006).

In contrast, in AD patients EEG abnormalities are typically represented by slowing of the background activity, which is reported either as widespread on the scalp derivations or as more prominent in temporal derivations (Valladeres-Neta et al., 1995).

In our previous systematic study (Bonanni et al., 2008), however, when attempting to differentiate EEG characteristics of DLB from those found in AD patients, the highest statistical yields were obtained in the comparison of dominant frequency and variability of the dominant frequency measured on recordings from posterior derivations. No statistical differences were found in temporal derivations between the 2 disease groups (Web material 1).

This finding can be explained by the presence of delta and/or theta activity in temporal derivations of both DLB and AD patients, in line with the results widely reported in literature (Babiloni et al., 2013).

Although AD patients present with an EEG pattern, characterized in posterior derivations by a dominant frequency in the alpha band prevalent in >55% of the analyzed EEG epochs and a dominant frequency variability <1.2 Hz, DLB patients present with derangement of EEG background activity in occipital derivations, characterized by dominant frequency in frequency bands lower than alpha (i.e., pre-alpha, theta, and delta) with dominant frequency variability >1.2 Hz and a frequency prevalence of pre-alpha in >40% of the analyzed EEG epochs and a frequency prevalence of alpha rhythm in <32%, as detailed in (Bonanni et al., 2008).

The aim of the present study is 2-fold: (1) to assess whether EEG characteristics described in our previous work as typical of early DLB and/or PDD are already present in MCI subjects; (2) evaluate whether possible EEG abnormalities in MCI individuals can predict the subsequent development of DLB.

## 2 Methods

### 2.1 Patients

The study sample was recruited among the new referrals in the year 2008 to the Memory Clinic and Movement Disorder Centre, Neurology Clinic of the University G. d’Annunzio of Chieti-Pescara, serving a population of 1,200,000 inhabitants of Abruzzo region, central Italy.

Given that the principal aim of the study was to assess the predictive value of EEG in the early diagnosis of DLB, we enriched our study sample by the inclusion of MCI subjects who had at least 1 core or suggestive DLB symptom (McKeith et al., 2005). Therefore, based on DLB prevalence in our dementia center (Bonanni et al., 2013) we selected 3 MCI subjects with 1 core or suggestive DLB symptom, according to DLB diagnostic criteria (McKeith et al, 2005) for every MCI subject without any DLB symptom.

A total of 99 subjects referred to our center because of subjective complaint of cognitive impairment. Forty-seven subjects were addressed to our attention by the general practitioners who had noticed changes in the cognitive performances of their patients. Concerns about cognition were expressed by the individual's family members in 30 cases and by the individuals themselves in the remaining 22 cases.

The individuals were categorized as MCI according to the criteria proposed by Petersen et al. (1999), including the presence of subjective complaint of memory dysfunction; pathologic scores in memory tests for age and educational level, underlying a memory impairment not interfering with the activities of daily living and normal general cognitive function.

International criteria for DLB diagnosis were applied to select MCI subjects with and without 1 core or suggestive feature of DLB.

Any subjects who had a prior history of PD or PD symptoms longer than 1 year before admission to the study were excluded from the study.

Furthermore, the international diagnostic criteria for AD (McKhann et al., 1984), vascular dementia (Roman et al., 1993), DLB (McKeith et al., 2005), and frontotemporal dementia (McKhann et al., 2001) were applied to exclude the presence of overt dementia.

All the subjects were tested with a composite battery of tests for cognition, including the Mini Mental State Examination (MMSE), the Clinical Dementia Rating (Morris et al., 1993), the Global Deterioration Scale (Reisberg et al., 1982), and the Dementia Rating Scale-2 (DRS-2) (Jurica et al., 2001).

An MMSE score of >24, a global Clinical Dementia Rating rating of stage 0.5 (defined as “questionable impairment”), a Global Deterioration Scale score of 2–3, and a DRS-2 score >123 were considered necessary to MCI diagnosis.

The presence of accompanying illnesses including neoplasia, blood hypertension, diabetes, obesity, malnutrition (vitamin deficiency), thyroidal diseases; alcohol present or past abuse, use of antidepressants, anticonvulsants, and benzodiazepines; depression as assessed by the Geriatric Depression Scale (Yesavage et al., 1982-1983) (score >5) were considered as exclusion criteria.

**Fig. 1:**
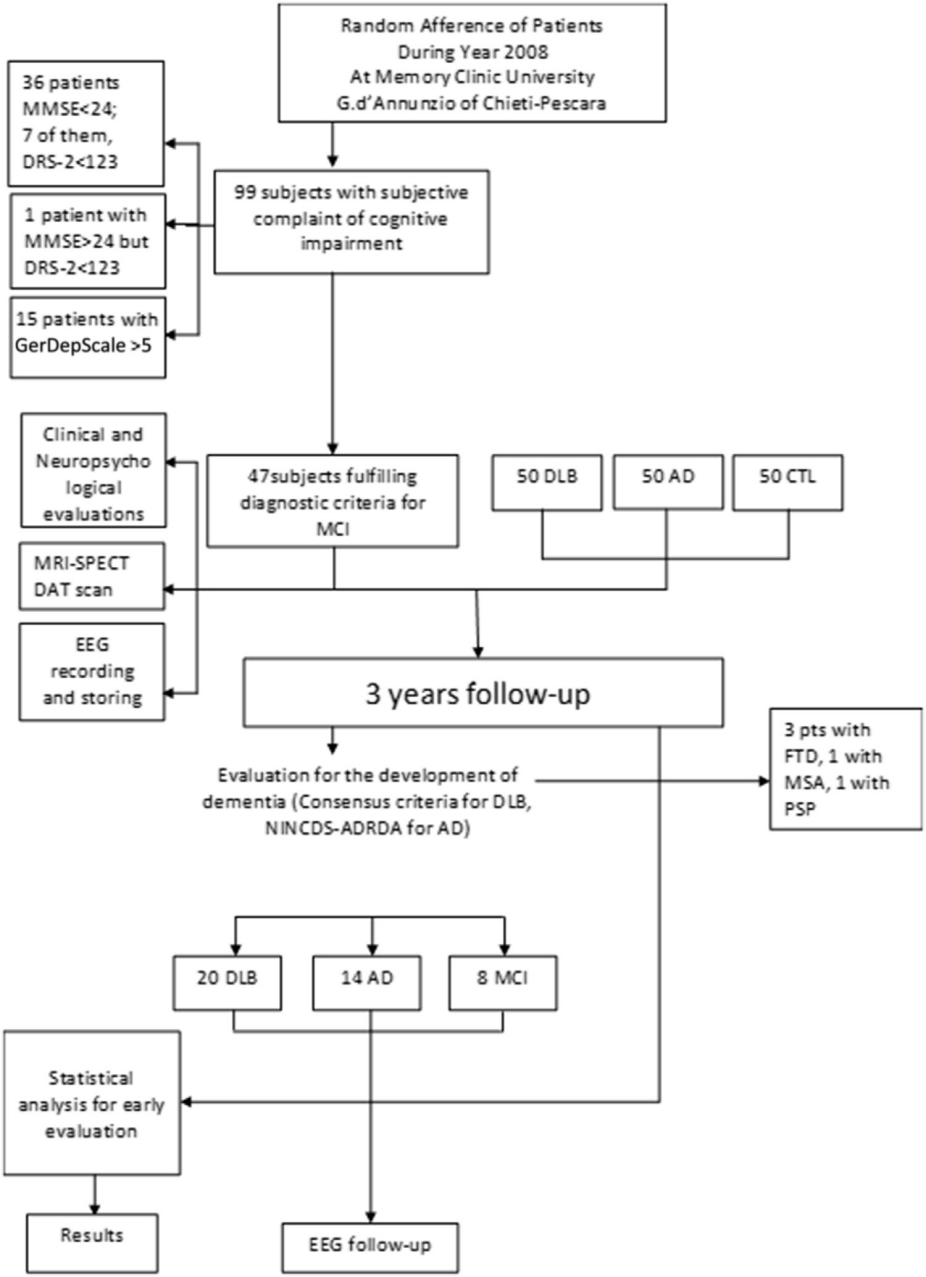
Study design flow chart. Abbreviations: AD, Alzheimer's disease; CTL, control subjects; DLB, dementia with Lewy bodies; DRS-2, Dementia Rating Scale-2; EEG, electroencephalogram; FTD, frontotemporal dementia; GerDepScale, Geriatric Depression Scale; MCI, mild cognitive impairment; MMSE, Mini Mental State Examination; MRI, magnetic resonance imaging; MSA, multiple system atrophy; PSP, progressive supranuclear palsy; SPECT-DAT scan, single photon emission computerized tomography-dopamine transporter scan.

A total of 47 of 99 individuals fulfilled the criteria for MCI and were admitted to the study. Among them 21 had 1 core or suggestive feature of DLB (Fig. 1 summarizes the study design).

For comparison 50 DLB and 50 AD patients in mild stage (disease duration: 1–2 years), matched with MCI subjects for age and educational level were randomly selected from our dementia register. The diagnosis of DLB or AD was made according to international criteria (McKeith et al., 2005, McKhann et al., 1984). In addition, 50 age and educational level-matched healthy individuals, with no either subjective or objective cognitive deficits and no evidence of any core or suggestive DLB symptoms, were recruited from our normative cohort and included in the study.

### 2.2 Study design

The study was composed of 2 parts:

• Cross-sectional study: clinical, neuropsychological, and EEG assessments were performed and analyzed in the 4 groups of subjects at admission to the study.
• Prospective longitudinal study: the 4 groups of subjects were followed for 3 years. Clinical and neuropsychological assessments were repeated every 6 months during the 3-year follow-up period.

Before being enrolled in the study, all subjects signed a written informed consent. The investigation was carried out according to the Declaration of Helsinki and subsequent revisions (Declaration of Helsinki, 1997).

All the subjects admitted to the study underwent a standardized neurologic examination, a magnetic resonance imaging scan, and a dopaminergic presynaptic ligand ioflupane single photon emission computer tomography (SPECT)-dopamine transporter (DAT) scan. The presence and severity of parkinsonian motor signs were rated through the motor part of the Unified Parkinson's Disease Rating Scale (Fahn and Elton, 1987).

The presence of REM sleep behaviour disorder (RBD) was evaluated according to minimal International Classification of Sleep Disorders criteria for RBD (World Health Organization, 1992) and confirmed by polysomnographic recordings.

The presence of behavioral and neuropsychiatric symptoms (including delusions, hallucinations, agitation/aggression, depression/dysphoria, anxiety, elation/euphoria, apathy, dysinhibition, irritability, and aberrant motor behavior) was rated with the Neuropsychiatric Inventory (Cummings et al., 1994).

The presence and severity of cognitive fluctuations were evaluated using the Clinician Assessment of Fluctuations (CAF) (Walker et al., 2000). The presence of frontal lobe dysfunction was assessed by Frontal Assessment Battery (Dubois et al., 2000).

For the longitudinal study, the conversion to AD was assessed in the MCI subjects and healthy individuals by the application of NINCDS-ADRDA criteria (McKhann et al., 1984), in the absence of any DLB core or suggestive symptoms (McKeith et al., 2005). The diagnosis of DLB was based on criteria recommended by the Consensus on DLB (McKeith et al., 2005).

Diagnosis of AD or non-AD (DLB) dementia was supported by the measurement of specific protein levels (Aβ_42_, tau, and Ptau) in the CSF (Parnetti et al., 2001).

A CSF AD profile was defined as follows: beta amyloid level <800 pg/mL, total tau >300 pg/mL, and phosporylated-tau protein >60 pg/mL.

A CSF profile suggestive of DLB was defined as follows: beta amyloid level <800 pg/mL, total tau >300 pg/mL, and P-tau <60 pg/mL (Parnetti et al., 2008).

All the clinical and neuropsychological examinations performed at admission to the study were repeated every 6 months until the end of the study in the 4 groups of subjects.

### 2.3 EEG recordings

The patients underwent EEG recordings according to previously published methods (Bonanni et al., 2008). Caffeine, nicotine, and alcohol were not allowed for at least 48 hours before neuropsychological and neurophysiological assessments. EEG recordings were analyzed with methods described in our previous work (Bonanni et al., 2008) by 2 experimenters unaware of the clinical conditions of the subjects. Briefly, quantitative EEGs (QEEGs) were recorded from 21 scalp derivations. Ag/AgCl disk scalp electrodes (19) were placed according to the international 10-20 system, EEG was recorded from Fp1, Fp2, Fz, F3, F4, F7, F8, Cz, C3, C4, Pz, P3, P4, T3, T 4, T5, T6, O1, and O2. Two additional electrodes were placed on A1 and A2. EEG activity was analyzed from single or multiple leads grouped to define the following scalp regions: anterior (Fz, Fp2, F7, Fp1, F3, F4, and F8), central (Cz, C3, and C4), posterior (Pz, P3, P4, O1, and O2), and temporal (T3, T4, T5, and T6). The posterior scalp region in our previous work was demonstrated to provide the highest statistical yield in the comparison of EEG traces between AD and DLB patients. Recordings were obtained with subjects resting comfortably, with their eyes closed. Patients' wakefulness was ascertained every 2 minutes inviting them to open their eyes and checking block reactions. A simultaneous electrooculogram was recorded and muscular or tremor artefacts were controlled with supplementary derivations. Two pairs of bipolar recording channels for respiration and electrocardiogram were also applied. EEG was acquired as a continuous signal for 30 minutes and visually inspected for current clinical interpretation or detection of artefacts and stored to be epoched in off-analysis setting as series of 2 seconds-long epochs.

The computer collected 10 minutes of EEG recorded with closed eyes, digitized at 1024 Hz with a low filter at 0.5 Hz and high filter at 70 Hz (decay constant 12 dB) with a 50 Hz notch filter in each channel. Blocks of artefact-free 2-second–long epochs appearing consecutively for 20–40 seconds were selected off-line by visual inspection after preprogrammed automatic blink reduction and muscle and tremor artefact rejection system and were compared with the remaining artefact-free epochs to avoid possible discrepancies among acquired sets. A total of 90 epochs per patient were processed by an automatic transforming program present in the NEUROSCAN SynAmps System performing a Fast Fourier Transform (FFT) on each epoch of EEG acquisition, allowing a frequency resolution = 0.5 Hz. The obtained spectra values were then processed to compute a mean power spectrum (mPS) for each channel and expressed in square μV (μV^2^). The mPS was divided automatically into 4 frequency bands (1–4 Hz [delta], 4–5.5 Hz [theta], 5.5–8 Hz [fast theta or pre-alpha], 8–12 Hz [alpha]). These bands were defined in our previous study (Bonanni et al., 2008).

Power spectrum was expressed as log-transformed FFT and dominant frequency (DF), that is, the frequency, where the spectral power was maximum, was evaluated for each epoch and for the mPS across all epochs. Mean relative power spectra (percentage of the global mPS for each frequency band) were computed and log transformed to normalize the data (Rodriguez et al., 1999). Mean relative power spectra was automatically calculated and expressed in numeric percentages for each one of the single epochs obtained from each scalp derivation.

Single channel power spectra were represented as compressed spectral arrays (CSA) showing the sequences of absolute or relative power spectra in each one of the 90 analyzed epochs.

CSA is the epoch to epoch representation of FFT, for each derivation. It shows peaks of amplitudes corresponding to frequencies in a single epoch (Bickford et al., 1973). These peaks of amplitude appear as salient patterns and peaks of amplitude that could either be relatively stable through time or change (i.e., different frequencies could have the highest amplitude through time). CSAs can be quantified by the following mathematical descriptors: (1) DF-dominant frequency; (2) DF range, expressing the range of dominant frequencies in the 90 epochs; (3) frequency prevalence (FP), that is, the percentage of epochs where prevalence of a dominant frequency band is observed (1%–100%); (4) band inscription (BI), that is, the percent of epochs where a peak of frequency is identified with a total amplitude above the mean amplitude of random peaks (noise); (5) frequency ratio, that is, band powers of pre-alpha or alpha versus delta, theta, and pre-alpha or alpha; (6) DF variability (DFV) expressing the variability of DF across the 90 analyzed epochs.

In our previous study the highest statistical yields between EEG characteristics of AD and DLB patients were obtained in the comparison of DF, DFV, and FP measured on recordings from posterior derivations.

FP showed that alpha was present in 60% or more epochs recorded in 100% of AD patients with an amplitude ratio of 8.0 ± 2.8 in comparison with every other frequency. In DLB patients, alpha was dominant in 32% or fewer epochs and absent in 66.7% of patients. Pre-alpha was prevalent in 40% or more epochs in 100% of DLB patients and in 11% or fewer epochs in 100% of AD patients.

Mean DF, DFV, and FP expressing the percentage of epochs where dominant alpha, pre-alpha, or theta-delta frequencies were found, and on the percentage of epochs where alpha, pre-alpha, theta-delta activities were detected (BI) and translated into 5 patterns of EEG activity classified in the 90 epochs recorded from derivations of AD and DLB patients.

The first pattern corresponded to dominant alpha in 60% or more of analyzed epochs (DF ≥8 Hz, FP alpha ≥60%), DFV of alpha below 0.6 Hz, mean DFV of all epochs below 1.6 Hz. BI of pre-alpha, theta, or delta activities below 30% of epochs; this pattern was defined as stable alpha, pattern 1.

The second pattern consisted of dominant alpha (≥8 Hz) in less than 50% of the epochs, mean DFV above 2 Hz, and dominant pre-alpha or theta (<8 Hz) in 40% of the epochs (FP pre-alpha >40%, BI of pre-alpha-theta-delta 50%); this pattern was defined as unstable alpha with pre-alpha or theta/delta, pattern 2.

The third pattern consisted of the absence of alpha, stable pre-alpha (DF ≤7.9 Hz) in 70% of more of analyzed epochs, DF range 5.6–7.9 Hz, DFV of the analyzed epochs below 1.0 Hz; this pattern was defined stable pre-alpha, pattern 3.

The fourth pattern consisted of the absence of alpha, dominant pre-alpha in less than 70% of the analyzed epochs, dominant theta or delta in 40% or more of epochs, DFV above 2.0 Hz; this pattern was defined unstable pre-alpha with theta and/or delta, pattern 4.

The last pattern consisted of absence of alpha, absence of alpha/pre-alpha dominant activity in more than 2 subsequent epochs with DFV above 4 Hz. This pattern was defined as unstable low frequency, pattern 5.

CSA sequences were classified as pattern 1 in 100% of the AD patients. EEG recordings of DLB were classified only in patterns 2, 3, 4, and 5, with a specificity and sensitivity of 100%.

Web material 1 reports pattern categorization according to the variables. In the present study, the EEG variables taken into consideration for traces analysis were as follows: CSA patterns (assuming for each pattern the aforementioned FP and BI), DF, and DFV recorded from posterior scalp derivations.

However, to reassure ourselves on the diagnostic validity of the categorization in EEG CSA specific patterns together with their mathematical descriptors, we also analyzed EEG traces according to different methods: classic interpretation method, EEG total, and relative power, mean frequency and mean frequency variability and CSA patterns with DF and DFV recorded also from anterior and temporal scalp derivations. Web material 1 shows results of the different EEG evaluation methods.

## 3 Statistical analysis

Differences between groups (MCI-AD, MCI-DLB, MCI non converters, AD, DLB, and controls) were tested using analysis of variance (ANOVA) with Bonferroni correction (checked with Kruskal-Wallis statistics) for continuous variables and Fisher exact test for categorical variables. As the main outcome, attempts were made to use polytomous logistic regression to test the differences across groups in each EEG characteristics adjusting for potential confounders. However, the presence of clear cutoffs, fully predicting the outcome for most EEG characteristics, made unfeasible any multivariate analysis that may produce estimates of the strength of the association between EEG patterns and type of disease.

To understand the relationship between the CSA at baseline and patients' condition at follow-up, we used correspondence analysis. This analysis is capable of providing the distance between the category points of 2 nominal variables in a 2-dimensional plot with similar categories plotted close to each other.

Correlations between EEG CSA patterns and neuropsychological test scores were tested with Spearman test. All analyses were carried out using STATA statistical software, version 9.0 (Stata Corp, Texas Station, TX, USA 2006).

## 4 Results

### 4.1 Cross-sectional study

#### 4.1.1 Clinical and neuropsychological assessment at admission

Demographic and neuropsychological test scores of the 4 groups of subjects at admission to the study are reported in Table 1. Table 2 reports the DRS-2 subitems scores for all the studied groups.

**Table 1:** Demographic, clinical, neuropsychological, laboratory characteristics of subjects included into the study

	MCI tot (47 pts)	MCI-C (34 pts)	MCI-NC (8 pts)	MCI-DLB (20 pts)	MCI-AD (14 pts)	DLB (50 pts)	AD (50 pts)	Controls (50 subjects)
Age	73.3 (7.8)	73.0 (8.4)	74.4 (5.8)	74.1 (7.0)	71.9 (9.7)	70.3 (4.9)	71.3 (4.4)	72.6 (5.4)
Gender	16 F/31 M	14 F/20 M	2 F/6 M	7 F/13 M	7 F/7 M	23 F/27 M	30 F/20 M	24 F/26 M
Education (y)	8.9 (4.7)	8.2 (5.1)	6.9 (3.8)	7.6 (5.3)	9.0 (4.8)	7.5 (5.5)	8.0 (5.8)	7.4 (6.2)
GerDepScale								
Onset	1.1 (1.3)	0.6 (0.9)	1.5 (1.5)	0.5 (0.9)	1.2 (1.3)	1.1 (1.1)	1.0 (1.1)	0.6 (0.7)
MMSE								
Onset	26.4 (2.0)	25.8 (1.6)	27.7 (1.8)	25.7 (1.1)	25.9 (1.8)	22.7 (1.3)	21.9 (2.5)	28.9 (0.8)
Follow-up	22.3 (3.7)	20.5 (3.2)	26.3 (1.0)	20.6 (3.2)	20.5 (3.3)	17.0 (3.2)	14.6 (3.5)	28.4 (0.9)
DRS-2								
Onset	123.2 (14.9)	120.0 (15.2)	130.0 (11.0)	120.5 (16.6)	119.6 (13.6)	102.9 (15.2)	100.3 (9.0)	137.3 (2.9)
Follow-up	111.4 (18.1)	105.0 (16.6)	126.5 (9.6)	106.6 (13.5)	103.1 (19.0)	82.9 (17.9)	73.7 (13.2)	135.7 (3.6)
FAB								
Onset	11.5 (3.8)	11.0 (3.7)	14.4 (3.9)	11.0 (3.8)	11.1 (4.0)	11.2 (1.6)	12.3 (2.1)	17.8 (0.5)
Follow-up	9.5 (3.9)	8.8 (3.6)	13.1 (4.3)	8.5 (3.9)	8.9 (3.8)	7.3 (2.3)	8.0 (3.3)	17.5 (0.9)
GDS								
Onset	2.5 (0.6)	2.5 (0.5)	2.1 (0.4)	2.6 (0.5)	2.7 (0.6)	4.0 (0.3)	4.2 (0.3)	0.0 (0.0)
Follow-up	3.5 (0.8)	3.9 (0.5)	2.3 (0.7)	4.4 (0.9)	4.5 (0.8)	4.4 (0.5)	4.5 (0.6)	0.0 (0.0)
CDR								
Onset	0.2 (0.3)	0.2 (0.2)	0.5 (0.0)	0.4 (0.2)	0.3 (0.2)	1.2 (0.5)	1.5 (0.5)	0.0 (0.0)
Follow-up	0.5 (0.5)	1.0 (0.2)	0.5 (0.0)	1.0 (0.3)	1.0 (0.1)	2.0 (1.0)	2.5 (1.5)	0.0 (0.0)
UPDRS								
Onset	8.2 (11.2)	6.8 (10.9)	7.1 (12.1)	10.8 (13.7)	2.9 (5.5)	12.3 (6.7)	0.4 (0.6)	0.0 (0.0)
Follow-up	13.6 (13.3)	13.8 (13.8)	9.5 (14.0)	23.0 (12.6)	4.3 (7.4)	16.9 (3.2)	0.8 (1.0)	0.0 (0.0)
CAF								
Onset	0.2 (0.8)	0.1 (0.7)	0.0 (0.0)	0.3 (1.1)	0.0 (0.0)	5.7 (2.1)	0.2 (0.2)	0.0 (0.0)
Follow-up	1.2 (2.0)	1.5 (2.2)	0.0 (0.0)	3.4 (2.0)	0.0 (0.0)	9.4 (1.4)	0.2 (0.2)	0.0 (0.0)
VH, % (no. of pts)								
Onset	2 (1)	2.9 (1)	0	0	7.1 (1)	28 (14)	2 (1)	0.0 (0.0)
Follow-up	26.5 (9)	26.5 (9)	0	40 (8)	7.1 (1)	64 (32)	4 (2)	0.0 (0.0)
Positive SPECT-DAT scan % (no. of pts)								
Onset	55.3 (26)	61.8 (21)	37.5 (3)	95 (19)	14.3 (2)	100	0	0
RBD % (no. of pts)								
Onset	40.4 (19)	47.1 (16)	25 (2)	70 (14)	14.3 (2)	64 (32)	2 (1)	2 (1)
Follow-up	48.9 (23)	58.8 (20)	25 (2)	85 (17)	21.4 (3)	94 (47)	2 (1)	2 (1)

When not differently stated, data are presented as mean (standard deviation).

Key: AD, Alzheimer's disease; CAF, Clinician Assessment of Fluctuations; CDR, Clinical Dementia Rating; CSF, cerebrospinal fluid; DLB, dementia with Lewy bodies; DRS-2, Dementia Rating Scale-2; FAB, Frontal Assessment Battery; GDS, Global Deterioration Scale; GerDepScale, Geriatric Depression Scale; MCI, mild cognitive impairment; MCI-AD, MCI subjects who converted to AD; MCI-DLB, MCI subjects who converted to DLB; MCI-C, MCI converted to AD or DLB; MCI-NC, MCI non converters; MMSE, Mini Mental State Examination; pts, patients; RBD, REM sleep behavior disorder; SPECT-DAT scan, single photon emission computer tomography-dopamine transporter; UPDRS, Unified Parkinson's Disease Rating Scale; VH, visual hallucinations.

**Table 2:** DRS-2 subitems scores in the studied groups

DRS2	MCI tot	MCI-C	MCI-NC	MCI-DLB	MCI-AD	DLB	AD	Controls
Attention								
Onset	34.2 (2.1)	33.6 (2.1)	35.8 (1.6)	33.1 (2.2)	34.5 (1.6)	24.4 (7.3)	28.1 (5.0)	35.0 (1.7)
Follow-up	31.2 (4.4)	30.4 (4.3)	34.4 (2.1)	29.9 (4.3)	31.2 (4.4)	18.1 (6.1)	20.5 (5.4)	34.4 (2.0)
Initiation perseveration								
Onset	31.1 (6.3)	29.9 (7.0)	33.8 (2.3)	30.0 (7.4)	29.7 (6.7)	26.8 (5.9)	26.4 (5.6)	34.3 (1.9)
Follow-up	27.1 (8.0)	26.2 (7.9)	32.8 (2.8)	26.2 (8.2)	26.4 (7.7)	22.7 (5.3)	19.1 (5.3)	33.9 (2.2)
Construction								
Onset	5.4 (1.0)	5.3 (1.1)	5.9 (0.4)	4.9 (1.3)	5.8 (0.4)	3.2 (1.8)	5.0 (1.4)	5.9 (0.3)
Follow-up	4.9 (1.2)	4.7 (1.2)	5.8 (0.7)	4.7 (1.4)	4.9 (0.9)	2.6 (1.3)	3.9 (1.1)	5.7 (0.7)
Conceptual								
Onset	33.7 (5.6)	32.7 (5.8)	35.3 (4.9)	32.3 (6.5)	33.4 (5.0)	25.8 (7.4)	25.6 (4.4)	37.4 (1.8)
Follow-up	30.8 (6.5)	29.8 (6.7)	34.9 (5.0)	30.3 (5.7)	29.1 (8.0)	21.7 (7.6)	18.9 (4.5)	37.1 (1.7)
Memory								
Onset	18.8 (5.1)	18.9 (5.4)	17.6 (5.2)	20.8 (5.5)	16.3 (4.0)	22.5 (4.0)	15.2 (4.3)	24.7 (0.5)
Follow-up	16.3 (5.4)	16.0 (5.7)	17.4 (5.3)	18.7 (5.1)	12.1 (4.3)	17.9 (4.8)	11.3 (3.7)	24.6 (0.6)
Total score								
Onset	123.2 (14.1)	120.4 (14.9)	128.3 (10.1)	121.0 (15.7)	119.6 (14.1)	102.6 (15.2)	100.3 (9.0)	137.3 (2.9)
Follow-up	110.3 (17.8)	107.1 (17.4)	125.1 (8.4)	109.6 (16.4)	103.6 (18.8)	82.9 (17.8)	73.7 (13.2)	135.7 (3.6)

Data are presented as mean (standard deviation).

Key: AD, Alzheimer's disease; DLB, dementia with Lewy bodies; DRS-2, Dementia Rating Scale-2; MCI, mild cognitive impairment; MCI-AD, MCI subjects who converted to AD; MCI-C, MCI converted to DLB or AD dementia; MCI-DLB, MCI subjects who converted to DLB; MCI-NC, MCI non converters; tot, total.

No differences were found, as for inclusion criteria, between the 4 groups with regard to age and educational level (Web table 1).

Among the 99 subjects referred to our clinic for the presence of subjective memory complaints, 52 subjects were excluded from the study. Among them, 36 subjects had an MMSE <24 (7 of them had <22) and 16 subjects had a Geriatric Depression Scale score >5 (mean score, 7.3 ± 1.0).

Our MCI population was therefore composed by 47 subjects: 31 males and 16 females. Demographic, neuropsychological, and laboratory characteristics of MCI subjects are reported in Table 1. Inter-groups differences were found in neuropsychological and clinical characteristics as summarized in Web table 1.

Twenty-two subjects had memory complaint and were classified as aMCI. Sixteen of them were single-domain aMCI, 6 had subjective impairment in more than 1 domain, including memory (executive function, attention), as assessed by DRS-2 scale.

Twenty-five subjects were affected by MCI involving other domains than memory (nonamnestic MCI, naMCI). Five of them were single-domain naMCI (with attention deficits or visuospatial disorder), 20 subjects were affected by multiple domain naMCI.

No differences were found in global performance at DRS2 between aMCI and naMCI (Web material 2). Twenty-one patients had parkinsonian signs at admission to the study (UPDRS motor score, 16.4 ± 10.8), with an onset within 6 months before the admission to the study.

The remaining 26 patients had an UPDRS motor score of 0–3. Nineteen MCI subjects presented with RBD at onset. Four subjects had a CAF score of 2–4. One patient had VH. Nineteen subjects presented with 1 core or suggestive clinical feature of DLB at admission to the study. Twenty-six subjects had a positive SPECT-DAT scan, showing caudate monolateral (7 patients) or bilateral hypocaptation.

#### 4.1.2 EEG characteristics at admission

The EEG recordings showed an EEG CSA pattern of 1 in 19 MCI subjects (DF = 9.6 ± 0.8, DFV 0.2 ± 0.3). Of them 15 subjects were aMCI (either single or multiple domain) and 4 subjects were naMCI (1 single domain). Twelve individuals showed an EEG CSA pattern 2 with a mean DF 8.2 ± 0.5 and DFV 1.7 ± 0.9. Of them 4 individuals were aMCI and 8 individuals were naMCI.

In 1 MCI subject, EEG recording was represented by an EEG CSA pattern 3 with DF of 7.5 and DFV of 0.0. Furthermore in 10 patients, EEG traces were characterized by a variable alpha dominant frequency with a mean DF of 9.1 ± 0.7 and DFV of 1.9 ± 0.7, as evidenced by the presence of peaks of frequency variable within the range of the alpha band (8.5–10.5 Hz). One of the patients was a single-domain aMCI. The remaining 9 patients were naMCI with 1 single-domain naMCI.

**Fig. 2:**
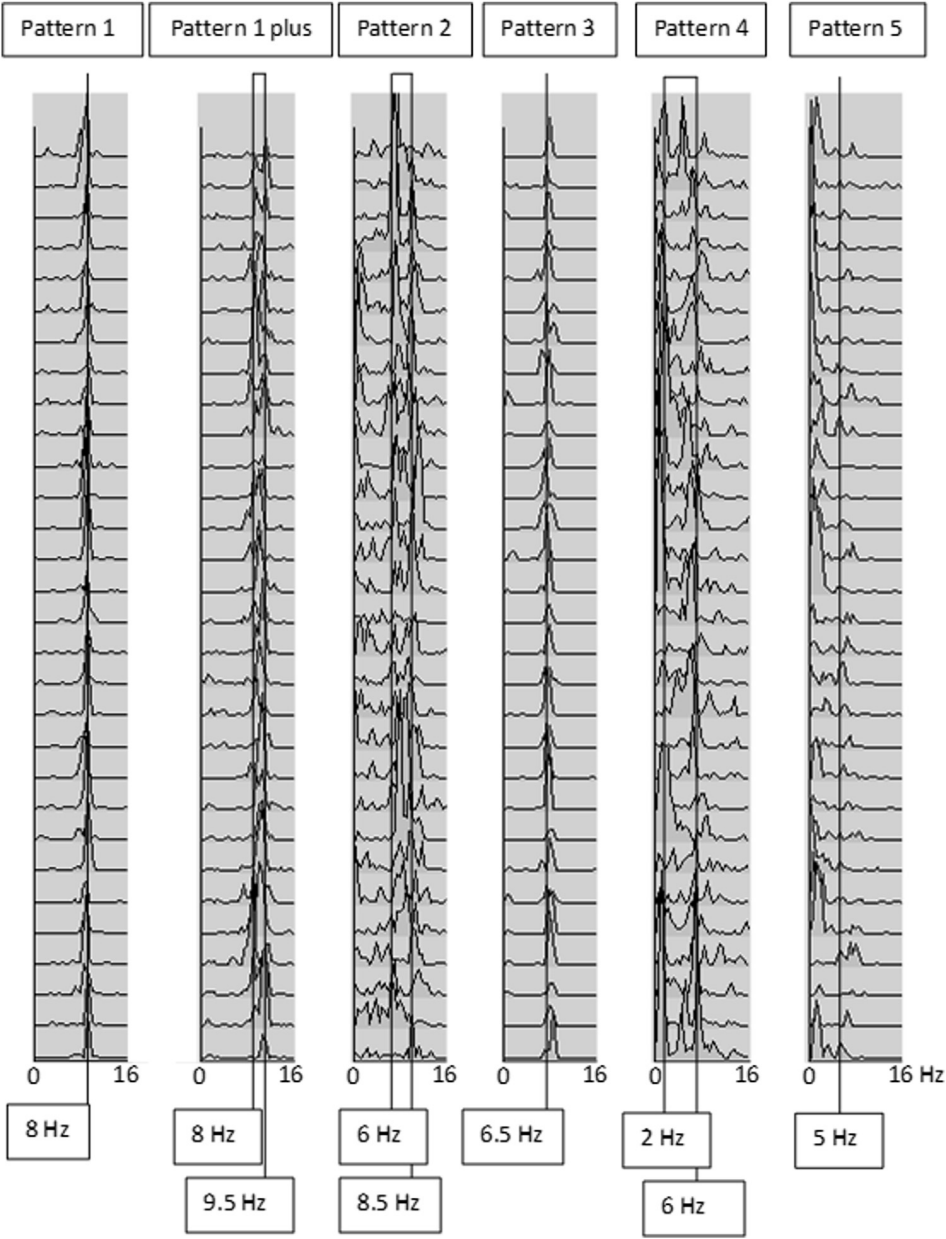
CSA of EEG recording from occipital derivations in 2 MCI patients (first 2 traces) and 4 DLB patients, showing the 6 EEG patterns described in the present study and in the previous EEG study (Bonanni et al., 2008). Pattern 1: dominant frequency (DF) in the alpha range with stable alpha frequency recorded at admission to the study in an MCI subject (later converted to AD). Pattern 1 plus: DF in the alpha frequency band with intrinsic variability = 1.5 Hz (DF variable between 8 and 9.5 Hz). This pattern was never observed in the previous EEG study (Bonanni et al., 2008) in patients with definite dementia. Pattern 2: notice variability of the dominant frequencies shifting from alpha (8.5 Hz) to dominant pre-alpha (6 Hz). Pattern 3: notice stable DF at 6.5 Hz. Pattern 4: DF variability, with the fastest frequency at 6 Hz and inscription of lower frequencies at 2 Hz. Pattern 5: degraded EEG pattern with the fastest frequencies slower than 5 Hz and delta activity at 1–4 Hz. Under each trace numbers in frames indicate the frequencies (in Hz) corresponding to power peaks. Abbreviations: AD, Alzheimer's disease; CSA, compressed spectral arrays; DLB, dementia with Lewy bodies; EEG, electroencephalogram; MCI, mild cognitive impairment.

This pattern, never observed in the cohort included in the previous EEG study (Bonanni et al., 2008), was characterized by DF in the alpha frequency band with intrinsic variability ≥1.5 Hz and was defined as “pattern 1 plus” (Fig. 2 shows the pattern 1 plus in comparison with examples of other CSA patterns). Table 3 summarizes CSA pattern distributions in MCI subjects together with values of the specific CSA mathematical descriptors (DF and DFV).

**Table 3:** EEG CSA patterns from posterior derivations in the different groups of subjects

	MCI-DLB (20)	MCI-AD (14)	MCI-NC (8)	DLB (50)	AD (50)	Controls (50)
CSA pattern 1						
Onset	0	13	5	0	50	50
DF	NA	9.7 (0.7)	9.8 (1.1)	NA	9.2 (1.3)	8.6 (1.0)
DFV	NA	0.1 (0.1)	0.1 (0.1)	NA	0.7 (0.3)	0.4 (0.3)
Follow-up	0	13	5	0	29	50
DF	NA	9.5 (0.6)	9.7 (0.9)	NA	8.4 (0.7)	8.5 (1.2)
DFV	NA	0.2 (0.3)	0.2 (0.2)	NA	1.0 (0.4)	0.4 (0.4)
CSA pattern 1 plus						
Onset	9	0	2	0	0	0
DF	9.5 (0.6)	NA	9.6 (0.6)	NA	NA	NA
DFV	1.8 (0.6)	NA	1.8 (0.3)	NA	NA	NA
Follow-up	0	0	1	0	0	0
DF	NA	NA	8.7	NA	NA	NA
DFV	NA	NA	1.0	NA	NA	NA
CSA pattern 2						
Onset	10	1	1	12	0	0
DF	8.4 (0.7)	9	7.5	7.8 (0.8)	NA	NA
DFV	1.8 (0.9)	1.5	2.5	2.7 (1.2)	NA	NA
Follow-up	12	1	2	2	11	0
DF	8.5 (0.7)	9	7.7 (0.5)	7.3 (0.4)	7.8 (0.5)	NA
DFV	2.2 (0.5)	1.5	2.4 (0.1)	4.3 (1.1)	2.1 (0.3)	NA
CSA pattern 3						
Onset	1	0	0	16	0	0
DF	7.6.	NA	NA	6.7 (0.6)	NA	NA
DFV	0	NA	NA	0.8 (0.6)	NA	NA
Follow-up	8	0	0	17	5	0
DF	7.8 (1.0)	NA	NA	6.9 (0.6)	6.8 (0.6)	NA
DFV	0.4 (0.3)	NA	NA	0.7 (0.4)	0.7 (0.4)	NA
CSA pattern 4						
Onset	0	0	0	15	0	0
DF	NA	NA	NA	4.3 (0.8)	NA	NA
DFV	NA	NA	NA	1.9 (0.5)	NA	NA
Follow-up	0	0	0	22	4	0
DF	NA	NA	NA	4.3 (0.8)	8.0 (0.0)	NA
DFV	NA	NA	NA	2.9 (0.5)	2.2 (0.3)	NA
CSA pattern 5						
Onset	0	0	0	7	0	0
DF	NA	NA	NA	3.0 (0.5)	NA	NA
DFV	NA	NA	NA	2.3 (0.7)	NA	NA
Follow-up	0	0	0	9	1	0
DF	NA	NA	NA	3.1 (0.6)	3.5	NA
DFV	NA	NA	NA	2.9 (0.4)	2.5	NA

Data are reported as mean (standard deviation).

Key: AD, Alzheimer's disease; CSA, compressed spectral array; DF, dominant frequency; DFV, dominant frequency variability; DLB, dementia with Lewy bodies; MCI, mild cognitive impairment; NA, not applicable.

### 4.2 Longitudinal study

#### 4.2.1 Clinical and neuropsychological evaluations at follow-up

At the end of the 3-year follow-up study, 34 subjects were affected by overt DLB or AD dementia (MCI converter). Their neuropsychological characteristics are reported in Table 1. Of them, 14 subjects diagnosed with dementia agreed to undergo lumbar puncture for the measurement of specific proteins levels in the CSF (Aβ_42_, tau, and P-tau) and 20 patients (19 subjects with a core and/or suggestive clinical DLB feature) fulfilled the criteria for DLB and were designated as MCI-DLB (i.e., former MCI subjects who converted to DLB). Of them 8 patients underwent lumbar puncture which showed a non-AD dementia protein pattern.

Two of them (10%) were aMCI at admission. Among the other 18 patients (90%), 4 were single-domain naMCI and 14 were multiple domain naMCI. Fourteen patients were diagnosed as AD and identified as MCI-AD (former MCI subjects who converted to AD). Among them, 6 patients underwent lumbar puncture which showed a protein pattern suggestive of AD. Twelve of the 14 patients (85.7%) were aMCI (9 single-domain aMCI) and 2 patients (13.3%) were multiple domain naMCI at admission to the study.

Three patients were diagnosed as affected by frontotemporal dementia (McKhann et al., 2001); 1 patient was diagnosed as affected by progressive supranuclear palsy (PSP) (Litvan et al., 1996); 1 patient was diagnosed as affected by multiple system atrophy (MSA) (Gilman et al., 1999).

At the end of follow-up, 8 patients still fulfilled the diagnostic criteria for MCI and were defined as MCI non converters and designated as MCI-NC. Among the MCI-NC, RBD was present in 2 subjects and was already present at admission to the study. UPDRS motor score did not change as compared with the baseline. These 2 subjects had a positive DAT-scan at admission to the study. Therefore, even if they were not fulfilling the diagnostic criteria for DLB, they showed features worthy to be followed for a longer follow-up.

Among the 8 MCI-NC, 1 subject, with aMCI, reverted to a normal subjective and objective cognitive performance, with an MMSE increase by 1 point. In 7 MCI-NC subjects, MMSE either was unchanged (2 patients) or decreased by 2 or points.

#### 4.2.2 EEG characteristics at follow-up

All of the 20 MCI subjects who received a diagnosis of probable DLB at follow-up (MCI-DLB), already presented with an EEG CSA pattern different than 1 at onset of the study (Table 3). EEG abnormalities described as CSA salient patterns >1 and with reduced DF and increased DFV were highly correlated with the appearance of fluctuating cognition at follow-up as assessed by CAF questionnaire (Spearman rho = 0.7, *p* = 0.001).

At follow-up, EEGs of MCI-DLB appeared more compromised as compared with their first EEG recording at admission to the study. None of the patients presented with an EEG CSA pattern 1 plus at follow-up (Table 3).

At admission to the study, 13 of the 14 MCI who converted to AD (MCI-AD) had an EEG CSA 1 with a mean DF of 9.7 ± 0.7 Hz and a mean DFV of 0.1 ± 0.1 Hz; and they maintained the same EEG CSA pattern 1 until the end of our follow-up, highlighting that AD patients are characterized by a stable alpha dominant activity in their EEG recordings (Bonanni et al., 2008) (Table 3).

The EEG in MCI-NC subjects was characterized by a CSA pattern 1 in 5 subjects and a CSA pattern >1 in the remaining 3 subjects (Table 3).

It was notable that the 2 MCI-NC subjects with a positive SPECT-DAT scan and with RBD had an EEG CSA different than 1 by the admission to the study.

### 4.3 Statistical comparisons

**Fig. 3:**
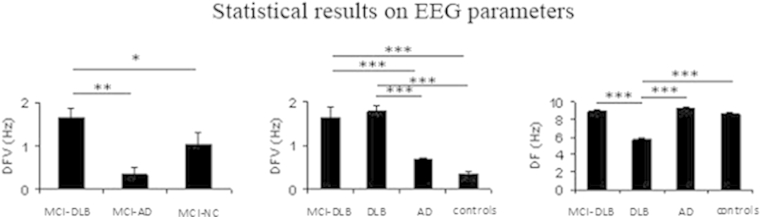
ANOVA results on EEG parameters at onset. (A) Statistical comparison on DFV among MCI groups: MCI-DLB, MCI-AD, and MCI-NC. (B) Statistical comparison on DFV among MCI-DLB, DLB, AD, and controls. (C) Statistical comparison on DF between MCI-DLB, DLB, AD, and controls; *p < 0.05, **p < 0.01, and ***p < 0.001. Abbreviations: AD, Alzheimer's disease; ANOVA, analysis of variance; DF, dominant frequency; DFV, dominant frequency variability; EEG, electroencephalogram; MCI, mild cognitive impairment; MCI-AD, MCI patients converted to AD at follow-up; MCI-DLB, MCI patients converted to DLB at follow-up; MCI-NC, MCI patients stable at follow-up.

ANOVA on MCI-DLB, MCI-AD and MCI-NC showed that DFV values of MCI-DLB were higher than MCI-AD (*p* < 0.01) and MCI-NC (*p* < 0.05) (Fig. 3). ANOVA on MCI-DLB, DLB, AD, and control subjects showed that at onset DF, DFV, and CSA were different across groups. DF was lower in DLB than in MCI-DLB (*p* < 10^−4^), AD (*p* < 10^−5^), and control subjects (*p* < 10^−5^), whereas DFV were higher in MCI-DLB and DLB than in AD and controls (*p* < 10^−5^) (Fig. 3).

**Fig. 4:**
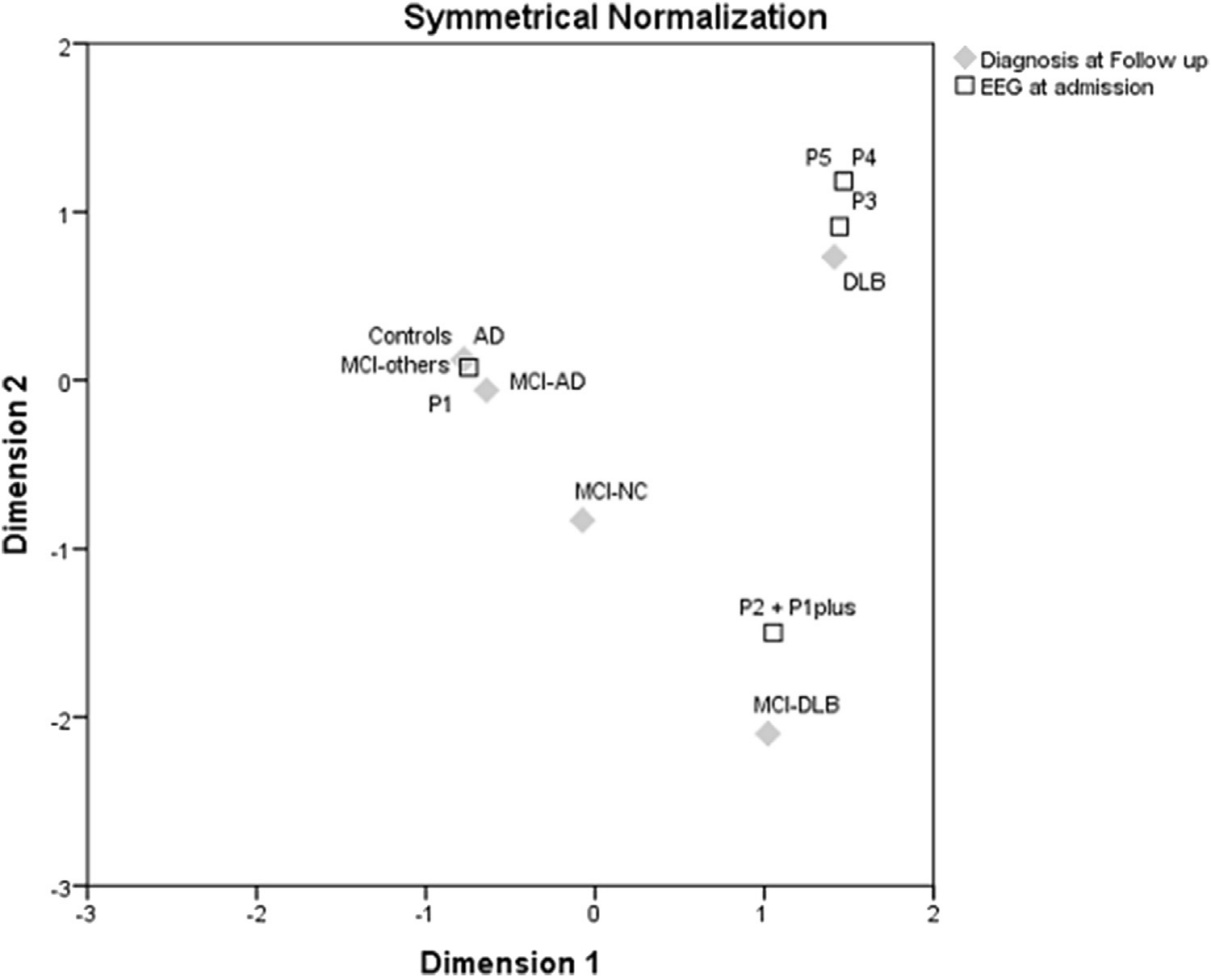
Correspondence analysis evidencing the relationship between the compressed spectral array (CSA) patterns at admission to the study and patients' condition at follow-up. Abbreviations: AD, Alzheimer's disease; DLB, dementia with Lewy bodies; EEG, electroencephalogram; MCI, mild cognitive impairment; MCI-AD, MCI subjects converted to AD at follow-up; MCI-DLB, MCI subjects converted to DLB at follow-up; MCI-NC, MCI subjects stable at follow-up; MCI-others, MCI subjects converted to other types of dementia; P1, EEG CSA pattern 1; P2, EEG CSA pattern 1 plus+ CSA pattern 2; P3, EEG CSA pattern 3; P4, EEG CSA pattern 4; P5, EEG CSA pattern 5.

When considering EEG classification according to CSA salient patterns, 100% of MCI-DLB had a CSA pattern >1 and 93% (13 of 14) of MCI-AD had a CSA pattern 1. Among the MCI-NC subjects 3 showed a DLB-like EEG pattern and 5 an AD-like EEG pattern. These results led to an overall predictive value for EEG CSA patterns of 76.2% in the 3-year follow-up study (Table 4). Fig. 4 shows the distribution in a 2-dimensional plot of the studied subjects according to EEG CSA patterns.

**Table 4:** Predictive value of EEG CSA salient patterns for the diagnosis of conversion to specific dementia

Observed	Predicted			
	MCI-DLB	MCI-NC	MCI-AD	Correct percentage
MCI-DLB	20	0	0	100.0
MCI-NC	3	0	5	0.0
MCI-AD	1	0	13	92.9
Overall percentage	54.8	0.0	45.2	76.2

Key: MCI-AD, mild cognitive impairment converted to Alzheimer's disease; MCI-DLB, mild cognitive impairment converted to dementia with Lewy bodies; MCI-NC, mild cognitive impairment non converters.

As shown in Fig. 4, MCI-AD, AD, and control subjects, all clustered close to EEG pattern 1. The MCI-DLB clustered close to EEG pattern 2 (including only in this figure, for clarity, both CSA pattern 1 plus and CSA pattern 2). In fact, it was evident at admission that these patients showed an initial deterioration of the oscillatory activity characterized by variable dominant frequency intrinsic to the alpha band or toward slower dominant frequencies (in the pre-alpha band). Finally, the MCI-NC tended to cluster between MCI-AD and MCI-DLB.

When considering CSA salient patterns as predictor variable of the neurologic condition at follow-up in a logistic regression, 100% of MCI-DLB had a CSA pattern >1, whereas 93% (13 of 14 patients) of MCI-AD had a CSA pattern 1. As already shown by the correspondence analysis, MCI-NC were not adequately classified, as part of the patients (5 of 8 patients) showed an MCI-AD-like CSA pattern while the remaining (3 of 8 patients) showed an MCI-DLB -like EEG.

Finally, considering that 19 MCI subjects already presented with 1 core or suggestive feature of DLB at admission, we compared the predictive value of CSA pattern at admission for the development of DLB at follow-up with the predictive value of the presence of early clinical features.

As reported in Table 5 and in Web material 3 the predictive value of the presence of DLB clinical features at the stage of MCI was lower than the predictive value of EEG abnormalities (Table 4).

**Table 5:** Predictive value of the presence of early DLB clinical features for the diagnosis of conversion to specific dementia

Classification				
Observed	Predicted			
	MCI-DLB	MCI-NC	MCI-AD	Percent correct
MCI-DLB	15	0	5	75.0
MCI-NC	2	0	6	0.0
MCI-AD	2	0	12	85.7
Overall percentage	45.2	0.0	54.8	64.3

Key: MCI-AD, mild cognitive impairment converted to Alzheimer's disease; MCI-DLB, mild cognitive impairment converted to dementia with Lewy bodies; MCI-NC, mild cognitive impairment non converters.

In particular, among the 19 subjects with 1 core and/or suggestive feature of DLB at admission, 15 converted to DLB at follow-up and 4 converted to AD. Furthermore, among the MCI-DLB, 5 patients did not present with any DLB clinical feature at admission (Table 5).

Web material 4 presents results on EEG in comparative groups and other dementias.

## 5 Discussion

The presence of EEG abnormalities in the very early phases of DLB dementia suggests that EEG could be used as a supportive diagnostic tool to study dementia subgroups (Bonanni et al., 2008).

In the present study, we tested the hypothesis that abnormalities of electrocortical arousal found in DLB patients could be already present at the stage of MCI and therefore could serve as a prodromal marker of subsequent development of DLB.

Our first finding was that, although control subjects showed a normal EEG recording with stable dominant alpha frequency, more than 50% of MCI subjects analyzed in our study showed EEG abnormalities, characterized by unstable dominant frequency inside the alpha band or between alpha and pre-alpha frequency bands, akin to abnormalities found in DLB patients in our previous EEG study (Bonanni et al., 2008).

The finding of increased DFV in the alpha range allowed the definition of a novel QEEG CSA pattern, never observed in our previous study on dementia patients, which we named CSA pattern 1-plus. It was characterized by intrinsic variability of alpha dominant frequency. In this pattern, alpha frequency, while still dominant in all the epochs explored, was unstable with increased mean dominant frequency variability (>1.5 Hz).

The appearance of this pattern, highly predictive of the subsequent development of cognitive fluctuations (rho = 0.7) suggested that disruption of electrocortical patterns is earlier and more subtle than the appearance of clinically assessed cognitive fluctuations (CAF questionnaire).

Cognitive fluctuations, although present across a range of dementias (Bradshaw et al., 2004, Ferman et al., 1999) are quantitatively and qualitatively different in DLB compared with, for example, AD appearing to be more driven by internal neurobiological processes such electrocortical variations (Bonanni et al., 2008, Bradshaw et al., 2004, Walker et al., 2000), in contrast to AD where such fluctuations are more dependent upon environmental and situational factors. Indeed cognitive fluctuations represent a core feature for the diagnosis of DLB and may be more specific to this condition in contrast to parkinsonian signs or visuospatial deficits (pentagon drawing) (Tiraboschi et al., 2006). However, the clinical assessment of fluctuations remains challenging (Lee et al., 2012). The CAF questionnaire which is perhaps the most widely used to assess the presence and severity of cognitive fluctuation has a cutoff score of 5 defined based on a patient population with moderate dementia (MMSE around 19). It is likely that such a score is too restrictive when assessing patient populations affected by mild dementia and even more when studying MCI subjects. Therefore, objective neurobiological measures of cognitive fluctuations are needed and we have previously argued for that fluctuations of EEG dominant activity could represent a more sensitive instrument to diagnose cognitive fluctuations (Bonanni et al., 2008).

Twenty (83.3%) MCI subjects presenting with abnormal EEGs (a QEEG pattern different than 1 with either a DF <8.0 or DFV >1.5), converted in the 3-year follow-up to DLB (the great majority, of which [17 patients, 85%] were naMCI). Assuming that a diagnosis of dementia depends upon the presence of a degree of global cognitive impairment (MMSE <24), and the presence of a functional impairment in activities of daily living (as widely accepted in literature) then 3 subjects (12.5%) with abnormal EEG were MCI non converters.

However, the MCI non converters with an EEG CSA different than 1 all had either a positive SPECT-DAT scan or presented with RBD. Thus, they could be tentatively classified as early or prodromal DLB. This possibility enhances the significance of our results, demonstrating that EEG may represent a powerful method to distinguish DLB with a 100% sensitivity and specificity when combined with other supportive features (McKeith et al., 2005).

If we look at the data retrospectively, among the 20 MCI patients converted to DLB dementia in the 3-year follow-up, 100% showed abnormal EEG findings at the stage of MCI. Among the 14 MCI converted to AD, only 1 patient had an abnormal EEG pattern at the stage of MCI.

Therefore, it is very likely that an MCI subject with the described EEG abnormalities (reduced DF and increased DFV defining EEG CSA patterns different than 1) who converts to dementia will convert to DLB.

When looking at the EEGs of the 5 patients who developed dementia in a form different than DLB or AD, none of these patients presented with abnormal EEG patterns. The correlation between oscillating EEG dominant frequency and fluctuating cognition is confirmed by the fact that none of the 5 patients suffered from cognitive fluctuations.

In keeping with the hypothesis that EEG abnormalities are linked to fluctuating cognition, we found that although those patients with MCI who converted to AD presented with the same EEG patterns found at admission to the study, in the MCI-DLB converter group EEG abnormalities became more severe and matched abnormalities found in DLB patient group at onset, correlating with increased prevalence and severity of cognitive fluctuations.

This suggests that, even if AD patients after 3 years of disease show EEG pattern disruption, as suggested by follow-up data in the 50 AD patients included in the study for comparison, these changes are modest as compared with DLB patients even at the stage of MCI. This implies that instability of electrocortical arousal is a prominent and early feature of DLB.

Our study has 2 main drawbacks: the presence of EEG normal patterns in AD patients, at least when mathematical strict quantitative methods are applied at onset of the disease (Bonanni et al., 2008), makes very unlikely that, even when applying standardized methods of quantitative EEG analysis, this will allow the prediction of conversion to AD given that in our study it was impossible to separate AD from healthy controls EEG patterns.

The current unfeasibility of using EEG as a predictive marker of Alzheimer's related dementia in MCI was recently underlined in a meta-analysis, which claimed that because of variability of patients populations included in diagnostic groups with variable odds ratios, sensitivity, and specificity of EEG analysis across the studies varied widely. It might be, therefore, that the greater EEG abnormalities observed by others in less recent “AD series” (Breslau et al., 1989, Coben et al., 1983, Hughes et al., 1989, Leuchter et al., 1993, Prinz and Vitiello, 1989, Rae-Grant et al., 1987) depend in part on heterogeneity of patient populations and, in particular, may reflect the inclusion of a variable number of DLB cases misdiagnosed as having AD. In support of this interpretation is the observation that in the most recent literature (Abásolo et al., 2006, Franciotti et al., 2006, Jeong, 2004, Kai et al., 2005, Mattia et al., 2003), EEG alterations reported for AD patients were modest and consistent with those shown in our study.

The second caveat comes from the notion that our patient population was selected in a tertiary clinic including both a dementia and a movement disorder center, therefore burdened by an ascertainment bias toward dementia associated with parkinsonian features (i.e., DLB) (Bonanni et al., 2013, Onofrj et al., 2010, Onofrj et al., 2013). Consequently, replication of our findings by other research groups are needed to validate quantitative EEG as a tool to predict the development of DLB.

## Disclosure statement

The authors have no conflicts of interest to disclose.

## Appendix A Supplementary data

Web material 1

Web material 2

Web material 3

Web material 4

Web table 1
