# Supplementary material for: Quantitative electroencephalogram utility in predicting conversion of mild cognitive impairment to dementia with Lewy bodies
Source: Neurobiol Aging. 2015 Jan;36(1):434–45. doi: 10.1016/j.neurobiolaging.2014.07.009 (PMC4270449; doi:10.1016/j.neurobiolaging.2014.07.009)
Supplement: Web material 1 [file mmc1.doc]

**Web material 1**

**Methods of EEG analysis**

Nineteen Ag/AgCl disk scalp electrodes, placed according to the international 10-20 system, recorded EEG from Fp1, Fp2, Fz, F3, F4, F7, F8, Cz, C3, C4, Pz, P3, P4, T3, T 4, T5, T6, O1, and O2. Two additional electrodes were placed on A1 and A2. EEG activity was analysed from single or multiple leads grouped to define the following scalp regions: anterior (Fz, Fp2, F7, Fp1, F3, F4, F8), central (Cz, C3, C4), posterior (Pz, P3, P4, O1, O2), temporal (T3, T4, T5, T6), peripheral (Fp1, Fp2, F8, T4, T6, O1, O2, T3, T5, Fz), and internal (F3, F4, Fz, C3, Cz, C4, P3, Pz, P4). Recordings were obtained with subjects resting comfortably, with their eyes closed. Patients’ wakefulness was ascertained every two minutes inviting them to open their eyes and checking block reactions. A simultaneous electrooculogram was recorded and muscular or tremor artefacts were controlled for with supplementary derivations. Two pairs of bipolar recording channels for respiration and electrocardiogram were also applied. EEG was acquired as a continuous signal for 30 min and visually inspected for current clinical interpretation or detection of artefacts and stored in order to be epoched in off-analysis setting as series of 2 seconds-long epochs. EEGs interpreted with classical visual inspection, corresponding to categories reported in previous literature [Barber PA et al, 2000; Briel RCG et al, 1999; Londos E et al, 2003] were defined as Classic Interpretation Methods (CIM) and reported in supplementary data 2. The computer collected 10 minutes of EEG recorded with closed eyes, digitised at 1024 Hz with a low filter at 0.5 Hz and high filter at 70 Hz (decay constant 12 dB) with a 50 Hz notch filter in each channel. Blocks of artefact-free 2 seconds-long epochs appearing consecutively for 20-40 sec were selected off-line by visual inspection after pre-programmed automatic blink reduction and muscle and tremor artefact rejection system and were compared with the remaining artefact-free epochs in order to avoid possible discrepancies among acquired sets. A total of 90 epochs per patient were processed by an automatic transforming program present in the NEUROSCAN SynAmps System performing a Fast Fourier Transform (FFT) on each second of EEG acquisition, allowing a frequency sensitivity=0.05 Hz. The obtained spectra values were then processed in order to compute a mean Power Spectrum (mPS) for each epoch and for each channel and expressed in square V (μV^2^). The mPS was divided automatically into 4 frequency bands (1-3.9 Hz [delta], 4-5.5 Hz [theta], 5.6-7.9 Hz [fast theta or pre-alpha], 8-12 Hz [alpha]). These bands were defined after the post hoc analysis with the purpose to facilitate identification of differences, in the description of results, as statistical differences were showed when theta band was halved in two parts (4-5.5 Hz, theta and 5.6-7.9 Hz pre-alpha).

Fast Fourier Transform-QEEG program expressed power values automatically after a log transform (log[x/(1-x)]) and indicated the Dominant Frequency (DF) of the entire power spectrum of each epoch, i.e. the specific frequency where the maximum power for a single epoch or a sum of multiple epochs was contained.

mean Relative Power Spectra (mRPS: percentage of the global mPS of each frequency band) were computed and log transformed [Rodriguez G, et al, 1999] to normalize the data, automatically calculated and expressed in numeric percentages for each one of the single epochs obtained from each scalp derivation. EEG power spectra were represented as scalp maps of band amplitudes measured on the 180 sec total analysis (Total Power) and analysed as Mean Frequency (MF), indicating the average frequency for the 90 epochs, and as mean frequency variability (MFV), representing changes of mean frequency during the 90 epochs. Single channel power spectra were also represented as Compressed Spectral Arrays (CSA) showing the sequences of absolute or relative power spectra in each one of the 90 analysed epochs.

CSA is the epoch to epoch representation of EEG FFT, for each derivation, it shows peaks of amplitudes, corresponding to frequencies in a single epoch [Bickford RG et al, 1973]. These peaks of amplitude appear as salient patterns. and peaks of amplitude could either be relatively stable through time or change (i.e. different frequencies could have the highest amplitude through time). CSAs can be quantified by the following mathematical descriptors: a) DF-dominant frequency, expressing the mean frequency where the maximum power was represented in the sum of the 90 epochs, b) DF range, expressing the range of dominant frequencies in the 90 epochs, c) Frequency Prevalence (FP), i.e. percent of epochs where prevalence of a dominant frequency band was observed (1.11%-100%), d) Band inscription, i.e. the percent of epochs where a peak of frequency was identified with a total amplitude above the mean amplitude of random peaks (noise), e) Frequency Ratio, i.e. band powers of pre-alpha or alpha vs delta, theta, pre-alpha or alpha, f) DF variability (DFV), expressing the variability of DF across the 90 analyzed epochs.

Statistics

Differences between groups (MCI-DLB, MCI-AD, AD, DLB, and controls) were tested using ANOVA with Bonferroni correction (checked with Kruskal Wallis statistics) for continuous variables; Fisher exact test for categorical variables. As the main outcome, attempts were made to use polytomous logistic regression to test the differences across groups in each EEG characteristics adjusting for potential confounders. However, the presence of clear cut-offs, fully predicting the outcome for most EEG characteristics, made unfeasible any multivariate analysis that may produce estimates of the strength of the association between EEG patterns and type of disease.

Significance Probability Mapping (SPM) based on t-test statistic [Duffy FH et al, 1981] was used to define regional differences between the scalp distribution of power of each frequency band for the different groups of patients. To investigate whether frontal, temporal and posterior EEG recordings produced different results, we used Wilkoxon matched-pairs signed-ranks test to compare the median of delta frequency pattern in AD patients resulting from the frontal derivation to the delta median in the same patients coming from temporal recordings. We used the same approach to test the difference between frontal and posterior derivation results, as well as temporal and posterior recordings, repeating all analyses for any other frequency pattern (theta, pre-alpha and alpha) in any group of patients.

The EEG variables MF, MFV, relative power, DF, DFV, FP from posterior derivations were included in a k-means cluster analysis (supplementary data 6) to verify results of compressed spectral arrays (CSA) visual analysis.

All analyses were carried out using STATA statistical software, version 9.0 (Stata Corp., Texas Station, TX, 2006).

**Results**

**Classic interpretation methods (CIM)**

Alpha activity with an approximate frequency of 8-12 Hz, responsive to eye-opening, was recorded from posterior derivations in all control, AD and MCI subjects. This activity was either regular, with fusiform sequences, in most of the control (84%) and AD subjects (75%), or intermittent and sporadically intermingled with faster frequencies in the remaining controls and some of the remaining AD patients. An intermittent alpha activity intermingled with irregular arrhytmic theta was also seen in other, few, AD patients, in 13 MCI subjects (31%) and in two thirds of DLB patients. In one MCI subjects and in the remaining third of DLB patients, alpha activity was absent and substituted by theta activity (6-7Hz), also responsive to eye-opening.

In anterior and temporal derivations, there were no differences among MCI, DLB and AD, with slow theta or delta frequencies observed in only 12% of DLB, 5% of AD patients, and arrhythmic sporadic theta observed in 2% of MCI subjects.

Neither did the groups differ in frontal or occipital intermittent delta activities, which were observed in only one AD, five DLB, and one MCI subject. Sharp transients in temporal derivations were present in only one AD and two DLB patients.

**Table 1a**. Percentage of patients presenting with the different EEG patterns evaluated through classic interpretation methods (CIM).

|  | **MCI (47)** | **MCI-DLB (20)** | **MCI-AD (14)** | **MCI-NC (8)** | **AD (50)** | **DLB (50)** | **Controls (50)** |
| --- | --- | --- | --- | --- | --- | --- | --- |
| Alpha frequency in posterior derivations ^a^ | 98 | 100 | 100 | 98 | 100 | 66 | 100 |
| Theta/delta in anterior- temporal derivations^b^ | 2 | 1 | 1 | 0 | 5 | 12 | 0.0 |
| Theta/delta in posterior derivations | 32 | 50 | 7 | 38 | 0 | 64 | 0.0 |
| Intermittent delta activity^b^ | 2 | 5 | 0 | 0 | 4 | 14 | 0.0 |
| Sharp transients^b^ | 0 | 0 | 0 | 0 | 4 | 4 | 0.0 |

^a^Alpha on posterior derivations is classified as unstable if faster (beta, >12 Hz) or slower (theta, 4-7.9) frequencies were detected. ^b^ Theta/delta, intermittent delta and sharp transients were positively reported when present on the traces, independently of relative predominance or sporadic appearance. MCI= Mild Cognitive Impairment, AD = Alzheimer Disease; DLB = Dementia with Lewy Bodies.

**Table 1b**. Comparison between percentages of MCI subjects converted to AD or DLB presenting with the different EEG patterns evaluated through classic interpretation methods (CIM) ^a^.

|  | MCI-AD vs MCI-DLB |
| --- | --- |
| Alpha frequency in posterior derivations ^a^ | *** |
| Theta/delta in anterior- temporal derivations^b^ | n.s. |
| Theta/delta in posterior derivations | *** |
| Intermittent delta activity^b^ | n.s. |
| Sharp transients^b^ | n.s. |

Asterisks mark P levels obtained with Chi-square test in the comparison between the two groups of subjects. * P< 0.05; ** P<0.01; *** P<0.001. N.S= not significant.

**EEG total and relative power**

Measurements of total powers showed differences only when significance probability mapping T-score statistics were applied, and only powers of a frequency band between 5.6 and 7.9 Hz were higher in DLB and MCI subjects converting to DLB in comparison with controls, AD subjects and MCI converting to AD (all P = 0.01–0.05). Therefore, the theta band was further separated into two

bands, slow theta (4.0–5.5 Hz) and fast theta or pre-alpha (5.6–7.9 Hz). Mean relative power spectra showed that the pre-alpha band amplitude was higher in MCI converting to DLB and DLB patients in comparison with control, AD and MCI converting to AD subjects (P>0.01) and that alpha band amplitude were higher in MCI converting to AD and AD patients compared with MCI converting to DLB and DLB subjects (P=0.05).

**Mean frequency and MFV**

Mean frequency values of the total 90 epochs were in the alpha range in controls, MCI converting to AD, and AD subjects and in the prealpha range in MCI converting to DLB and DLB subjects. The EEG mean frequency value separated controls from all patient groups (P<0.05). Table 3 shows mean frequencies. Mean frequencies on scalp could be analysed on each single epoch, thus showing the MFV. In 50% of MCI subjects (all MCI converting to DLB and 7 out of 13 MCI non converter) and in 75% of DLB mean frequency varied across time, with erratic representation of frequencies in the theta/pre-alpha and alpha range in posterior derivations of the two hemispheres. 13 of the 14 MCI subjects converting to AD, 5 MCI non converter and all the AD patients showed a stable pattern of mean frequency across the 180 s period, predominantly in the alpha range. Table 2 shows results of mean relative power spectra.

Table 2a

Relative power spectra from all derivations (global mRPS), Mean Frequency (MF) and MF variability (MFV) at admission to the study.

|  |  | **MCI-DLB**  **(20)** | **MCI-AD (14)** | **MCI-NC (8)** | **AD (50)** | **DLB (50)** | **Controls (50)** |
| --- | --- | --- | --- | --- | --- | --- | --- |
| **Q relative** | **delta (1.0-3.9Hz)** | 8.8 (1.9) | 8.2 (2.0) | 8.5 (2.0) | 10.1 (4.7) | 11.2 (4.8) | 8.2 (1.5) |
|  | **theta (4.0-5.5Hz)** | 20.0 (6.5) | 7.2 (7.1) | 11.2 (7.0) | 10.7 (3.5) | 23.2 (11.7) | 7.2 (2.0) |
|  | **Pre-alpha **  **(5.6-7.9Hz)** | 33.1 (12.2) | 5.7 (1.0) | 12.4 (3.6) | 9.1 (2.5) | 41.4(13.9) | 8.3 (1.4) |
|  | **alpha (8.0-12.0Hz)** | 22.2 (10.6) | 69.1 (3.1) | 35.1 (11.0) | 70.1 (4.3) | 24.2 (10.0) | 76.3 (2.3) |
| **MF** |  | 7.5 (2.5) | 8.0 (1.1) | 8.0 (2.0) | 8.0 (1.2) | 7.1 (2.2) | 8.1 (1.4) |
|  |  |  |  |  |  |  |  |
| **MFV** |  | 1.8 (1.0) | 1.1 (1.0) | 1.2 (0.9) | 1.2 (0.8) | 1.9 (1.1) | 1.0 (0.4) |

Values are reported as mean (standard deviation). MCI= Mild Cognitive Impairment; AD = Alzheimer Disease; DLB = Dementia with Lewy Bodies.

Table 2b. Statistical comparison of relative power spectra from all derivations (global mRPS), Mean Frequency (MF) and MF variability (MFV) at admission to the study between MCI subjects converted to AD (MCI-AD) or DLB (MCI-DLB).

|  |  | MCI-DLB vs. MCI-AD |
| --- | --- | --- |
| **Q relative** | **delta (1.0-3.9Hz)** | n.s. |
|  | **theta (4.0-5.5Hz)** | n.s. |
|  | **Pre-alpha (5.6-7.9Hz)** | * |
|  | **alpha (8.0-12.0Hz)** | ** |
| **MF** |  | * |
|  |  |  |
| **MFV** |  | *** |

Asterisks mark P levels obtained with T test in the comparison between MCI-DLB and MCI-AD subjects. * P< 0.05; ** P<0.01; *** P<0.001. N.S= not significant.

**Compressed spectral array (CSA).**

Table 3 shows DF, DFV, DF range, FP from grouped derivations of patients and controls.

The highest statistical yields were obtained in the comparison of DF, DFV and FP measured on recordings from posterior derivations (MCI-AD vs MCI-DLB p<0.001).

FP showed that alpha was present in 60% or more epochs recorded in 93% of MCI-AD subjects and 100% of AD patients with an amplitude ratio of 8.1±2.9 in comparison with every other frequency. Alpha was dominant in less than 40% of epochs in all MCI-DLB and DLB patients. Pre-alpha was prevalent in 40% or more epochs in 53% of MCI-DLB and 100% of DLB patients and in 11% or fewer epochs in 93% of MCI-AD and 100% of AD patients.

Pre-alpha/alpha band power ratio (mean band power ratio from all scalp derivations) was 3.0±3.5 in DLB and 0.9±0.8 in MCI-DLB, 0.1±0.03 in AD, 0.2±0.01 in MCI-AD (MCI-DLB vs MCI-AD, p=0.01).

Based on mean Dominant Frequency (DF), DF Variability (DFV), on Frequency Prevalence (FP) expressing the percentage of epochs where dominant alpha, pre-alpha, or theta-delta frequencies were found, and on the percentage of epochs where alpha, pre-alpha, theta-delta activities were detected (Band Inscription, BI), five patterns of EEG activity could be classified in the 90 epochs recorded from each derivations of patients or controls.

The first pattern corresponded to dominant alpha in 60% or more of analyzed epochs (DF≥8 Hz, FP alpha ≥60%), DFV of alpha below 0.6 Hz, mean DFV of all epochs below 1.5 Hz, Band Inscription of pre-alpha, theta or delta activities below 30% of epochs: this pattern could be defined Stable alpha, Pattern 1.

The second pattern consisted of dominant alpha (≥8 Hz) in less than 50% of epochs, mean DFV above 2 Hz, dominant pre-alpha or theta (<8 Hz) in 40% of more of epochs (FP pre-alpha >40%, BI of pre-alpha-theta-delta 50%): this pattern was defined unstable alpha with pre-alpha or theta/delta, pattern 2.

The third pattern consisted of absence of alpha, stable pre-alpha (DF≤7.9 Hz), in 70% of more of analysed epochs, DF range 5.6-7.9 Hz, DFV of the analysed epochs below 1.0 Hz; this pattern was defined stable pre-alpha, pattern 3.

The fourth pattern consisted of absence of alpha, dominant pre-alpha in less than 70% of analysed epochs, dominant theta or delta in 40% or more of epochs, DFV above 2.0 Hz; this pattern was defined unstable pre-alpha with theta/delta, pattern 4.

The last pattern consisted of absence of alpha, absence of alpha/pre-alpha dominant activity in more than 2 subsequent epochs with DFV above 4 Hz. This pattern was defined as unstable low frequency, pattern 5.

A sixth pattern, never observed in our previous EEG study (Bonanni et a, 2008) consisted of dominant frequency in the alpha band with intrinsic variability ≥ 1.5 Hz and was defined pattern 1-plus.

Table 4 summarizes the 6 patterns.

| **CSA**  **Patterns** | **DF (Hz)** | **DFV (Hz)** | **FP (% of epochs)** | **BI (% of epochs)** |
| --- | --- | --- | --- | --- |
| **1**  **(stable alpha)** | Alpha  (8.0-12.0) | <1.5 | Alpha in >60% | Pre-alpha/theta/delta in <30% |
| **1 plus**  **(unstable alpha)** | Alpha  (8.0-12.0) | >1.5 | Alpha in >60% | Pre-alpha in < 20% |
| **2**  **(unstable alpha+pre-alpha)** | Alpha  (8.0-12.0)  Pre-alpha  (5.6-7.9) | >2 | Alpha in < 50%  Pre-alpha in >40% | Pre-alpha/theta/delta in 50% |
| **3**  **(stable pre-alpha)** | Pre-alpha  (5.6-7.9) | <1.0 | Pre-alpha in >70% | Theta in <30% |
| **4**  **(unstable pre- alpha+theta/delta)** | Pre-alpha  (5.6-7.9)  Theta/delta  (1.0-5.5) | >2.0 | Pre-alpha in <70%  Theta/delta in >40% | Pre-alpha/theta/delta in 50% |
| **5**  **(unstable theta or delta)** | Theta/delta  (1.0-5.5) | >4 | Theta/delta in >80% | Theta/delta in 50% |

CSA sequences were classified as pattern 1 in 100% of controls, and in 100% of recordings from posterior derivations of AD patients and 93% of MCI-AD. CSA sequences in DLB were classified only in patterns 2, 3, 4, 5. CSA sequences in MCI-DLB were classified in patterns 1 plus and 2.

Table 5 summarizes results.

**Table 5A Dominant frequency (DF), DF variability (DFV), DF range, frequency prevalence (FP)/Band inscription (BI) for each frequency band, CSA patterns from frontal, temporal, parieto-occipital derivations from EEGs recorded at admission to the study.**

|  | EEG variables | **MCI-DLB (20)** | **MCI-AD (14)** | **MCI-NC (8)** | **AD (n=50)** | **DLB**  **(n= 50)** | **Controls**  **(n= 50)** |
| --- | --- | --- | --- | --- | --- | --- | --- |
|  | **DF** | 8.0 (2.0) | 8.0 (1.0) | 8.0 (1.0) | 8.1 (1.3) | 7.0 (1.6) | 9.0 (1.2) |
|  | **DFV** | 2.0 (1.2)) | 1.2 (0.9) | 1.5 (1.0) | 1.6 (1.3) | 2.8 (1.6) | 0.5 (0.3) |
|  | **FP/BI** |  |  |  |  |  |  |
| **F** | **Alpha** | 24 (7) / 30 (9) | 70 (9) / 76 (8) | 51 (6) / 52 (9) | 70 (8) / 76 (3) | 25 (11) / 35 (15) | 86 (3) / 90 (2) |
|  | **Pre-alpha** | 51 (9) / 54 (9) | 10 (5) / 13(6) | 40 (8) / 45 (7) | 8 (3) / 11 (5) | 53 (10) / 75 (11) | 6 (2) / 6 (2) |
|  | **Theta** | 8 (3) / 10 (4) | 8 (3) / 9 (3) | 7 (3) / 9 (4) | 13 (3) / 16 (6) | 8 (4) / 11 (4) | 4 (1) / 5 (2) |
|  | **Delta** | 3 (1) / 4 (1) | 2 (1) / 3 (1) | 2 (2) / 3 (3) | 8 (5) / 10 (5) | 10 (4) / 10 (4) | 3 (2) / 3 (3) |
|  | **CSA pattern**  **1-stable alpha**  **1 plus- variable alpha**  **2-unstable alpha+ pre- alpha**  **3-stable pre- alpha**  **4-unstable pre alpha+theta/delta**  **5-unstable theta or delta** | 0  45  40  0  15  0 | 71  0  29  0  0  0 | 37.5  25  25  12.5  0  0 | 80  0  5  5  5  5 | 0  0  25  33.3  30.6  11.1 | 100  0  0  0  0  0 |
|  |  |  |  |  |  |  |  |
|  | **DF** | 8.0 (1.8) | 8.0 (1.0) | 8.0 (1.3) | 8.0 (0.8) | 6.8 (1.6) | 8.7 (1.1) |
|  | **DFV** | 2.0 (1.1)) | 1.2 (0.9) | 1.5 (1.1) | 1.3 (0.6) | 1.9 (1.2) | 0.4 (0.3) |
|  | **FP/BI** |  |  |  |  |  |  |
| **T** | **Alpha** | 25 (7) / 31 (9) | 70 (9) / 77 (7) | 52 (6) / 57 (9) | 69 (5) / 75 (6) | 24 (7) / 29 (8) | 87 (3) / 90 (3) |
|  | **Pre-alpha** | 50 (9) / 54 (9) | 7 (3) / 10 (6) | 40 (8) / 45 (7) | 8 (3) / 10 (5) | 54 (8) / 60 (9) | 4 (2) / 4 (2) |
|  | **Theta** | 8 (3) / 10 (4) | 8 (3) / 9 (3) | 7 (3) / 9 (4) | 13 (2) / 16 (3) | 8 (4) / 10 (5) | 3 (2) / 3 (2) |
|  | **Delta** | 3 (1) / 4 (1) | 2 (1) / 3 (1) | 2 (2) / 3 (3) | 8 (3) / 8 (3) | 9 (6) / 10 (6) | 5 (1) / 5 (1) |
|  | **CSA pattern**  **1-stable alpha**  **1 plus- variable alpha**  **2-unstable alpha+ pre- alpha**  **3-stable pre- alpha**  **4-unstable pre alpha+theta/delta**  **5-unstable theta or delta** | 0  47  40  0  13  0 | 71  0  29  0  0  0 | 37.5  25  25  12.5  0  0 | 80  0  5  5  5  5 | 0  0  25  33.3  30.6  11.1 | 100  0  0  0  0  0 |
|  |  |  |  |  |  |  |  |
|  | **DF** | 8.1(0.5) | 9.7 (0.2) | 9.1 (1.0) | 8.3 (0.6) | 7.4 (1.6) | 8.6 (1.0) |
|  | **DFV** | 2.0 (0.1) | 0.2 (0.4) | 0.9 (0.8) | 1.1 (0.4) | 1.8 (1.2) | 0.4 (0.3) |
|  | **FP/BI** |  |  |  |  |  |  |
|  | **Alpha** | 24 (8) / 25 (6) | 72 (7) / 70 (6) | 55 (7) / 58 (9) | 74 (6) / 75 (8) | 19 (5) / 20 (7) | 86 (3) / 89 (4) |
| **P** | **Pre-alpha** | 60 (9) / 62 (10) | 5 (2) / 4 (1) | 40 (5) / 44 (1) | 6 (3) / 8 (3) | 61 (8) / 63 (8) | 5 (2) / 5 (3) |
|  | **Theta** | 8 (4) / 10 (5) | 7 (2) / 8 (2) | 7 (2) / 9 (4) | 11 (4) / 11 (5) | 10 (4) / 11 (5) | 3 (2) / 3 (2) |
|  | **Delta** | 3 (3) / 3 (3) | 3 (2) / 3 (3) | 3 (2) / 3 (3) | 6 (2) / 6 (3) | 7 (3) / 7 (3) | 3 (1) / 3 (2) |
|  | **CSA pattern**  **1-stable alpha**  **1 plus- variable alpha**  **2-unstable alpha+ pre- alpha**  **3-stable pre- alpha**  **4-unstable pre alpha+theta/delta**  **5-unstable theta or delta** | 0  45  50  5  0  0 | 93  0  7  0  0  0 | 62.5  25  12.5  8  0  0 | 100  0  0  0  0  0 | 0  0  33.3  25.0  30.6  11.1 | 100  0  0  0  0  0 |

F= frontal derivations; T= temporal derivations; P= parieto-occipital derivations. DF= Dominant frequency ; DFV= dominant frequency variability; FP= frequency prevalence; BI= Band Inscription; CSA= Compressed Spectral Array. DF, DFV and DF range are expressed in Hz; FP and BI are expressed as mean (standard deviation) and are approximated to the unit; CSA patterns are expressed as percentage of patients for each group. MCI= Mild Cognitive Impairment; AD = Alzheimer Disease; DLB = Dementia with Lewy Bodies.

Table 5B. Statistical comparisons of the EEG variables presented in table 4A between MCI-DLB and MCI-AD subjects.

|  | EEG variables | **MCI-DLB vs. MCI-AD** |
| --- | --- | --- |
|  | **DF** | ** |
|  | **DFV** | *** |
|  | **FP/BI** |  |
| **F** | **Alpha** | *** |
|  | **Pre-alpha** | *** |
|  | **Theta** | *** |
|  | **Delta** | n.s. |
|  |  |  |
|  | **DF** | *** |
|  | **DFV** | * |
|  | **FP/BI** |  |
| **T** | **Alpha** | *** |
|  | **Pre-alpha** | *** |
|  | **Theta** | *** |
|  | **Delta** | n.s. |
|  |  |  |
|  | **DF** | *** |
|  | **DFV** | *** |
|  | **FP/BI** |  |
|  | **Alpha** | *** |
| **P** | **Pre-alpha** | *** |
|  | **Theta** | n.s. |
|  | **Delta** | n.s. |

F= frontal derivations; T= temporal derivations; P= parieto-occipital derivations. DF= Dominant frequency; DFV= dominant frequency variability; FP= frequency prevalence; BI= Band Inscription.
