# Supplementary material for: Quantitative electroencephalogram utility in predicting conversion of mild cognitive impairment to dementia with Lewy bodies
Source: Neurobiol Aging. 2015 Jan;36(1):434–45. doi: 10.1016/j.neurobiolaging.2014.07.009 (PMC4270449; doi:10.1016/j.neurobiolaging.2014.07.009)
Supplement: Web material 2 [file mmc2.doc]

Web material 2


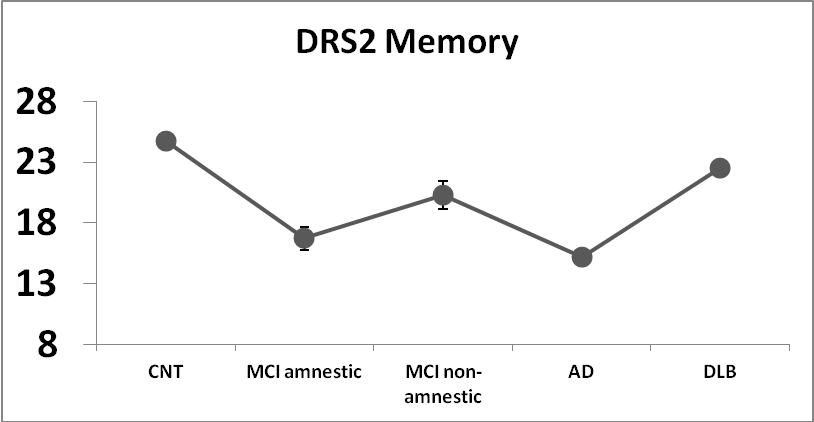


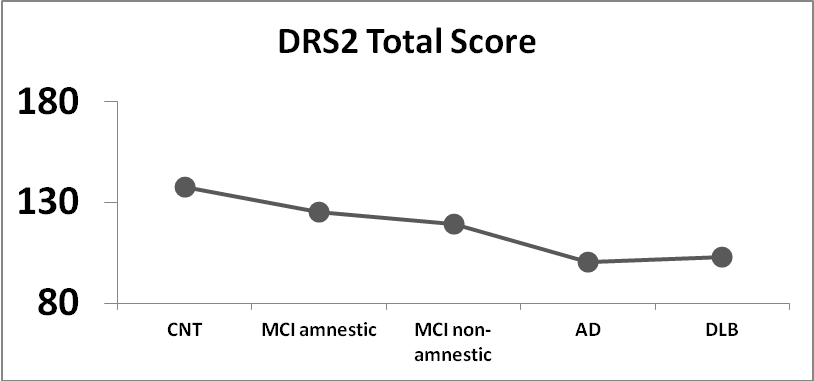


The graphs show that the amnestic and non amnestic MCI groups have similar global performance at the DRS2 (post hoc, p=1) but differ in terms of memory impairments (post hoc, p=0.03). In addition, they do not show a clear dementia (total raw scores are greater in MCI than AD and DLB; MCI amnestic vs. AD post hoc, p<0.001; MCI amnestic vs. DLB post hoc, p<0.001; MCI non-amnestic vs. AD post hoc, p<0.001; MCI non-amnestic vs. DLB post hoc, p<0.001;) but have memory or other cognitive impairments (they show poor performance in comparison to the control group; MCI amnestic vs. CNT post hoc, p=0.001; MCI non-amnestic vs. CNT post hoc, p<0.001).
