# Supplementary material for: Quantitative electroencephalogram utility in predicting conversion of mild cognitive impairment to dementia with Lewy bodies
Source: Neurobiol Aging. 2015 Jan;36(1):434–45. doi: 10.1016/j.neurobiolaging.2014.07.009 (PMC4270449; doi:10.1016/j.neurobiolaging.2014.07.009)

Web material 3

We report the graphs showing the neuropsychological profile of MCI subjects divided on the basis of the presence/absence of DLB clinical features at admission to the study and at follow-up.

Anova for repeated measure (10X2) was performed, where the factors were neuropsychological test scores (ten levels) and time (two levels). The presence/absence of clinical features was the between factor. The interactions of interest were testXDLB feature (F=1.163, P=0.317) and testXtimeXDLB feature (F=0.352, p=0.95). No differences are evidenced between the two groups of subjects.


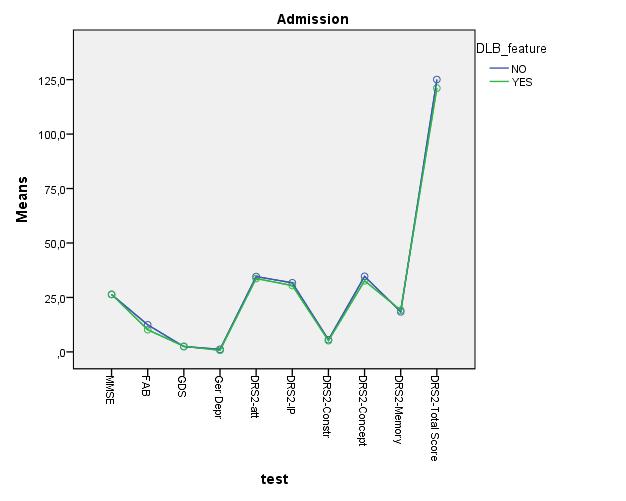


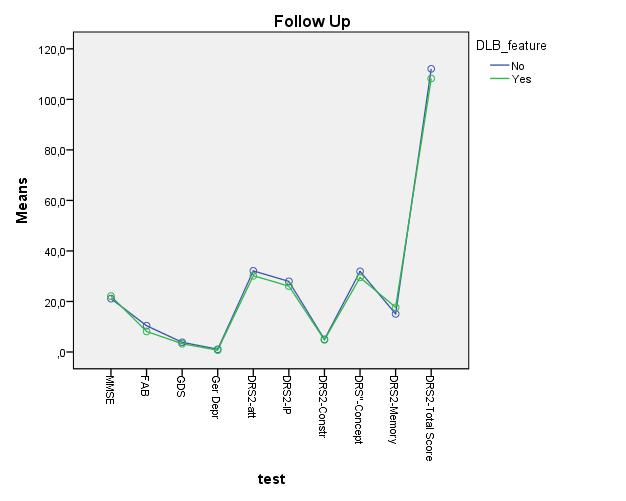

Supplement: Web material 3 [file mmc3.doc]
