# Supplementary material for: Quantitative electroencephalogram utility in predicting conversion of mild cognitive impairment to dementia with Lewy bodies
Source: Neurobiol Aging. 2015 Jan;36(1):434–45. doi: 10.1016/j.neurobiolaging.2014.07.009 (PMC4270449; doi:10.1016/j.neurobiolaging.2014.07.009)
Supplement: Web material 4 [file mmc4.doc]

**Web material 4**

**EEG in comparative groups and other dementias**

EEG characteristics in the comparative groups are reported in table 2. Consistent with our previous findings, none of the DLB patients had an EEG CSA pattern 1 at admission. 12 DLB patients had an EEG CSA pattern 2, 16 had a CSA pattern 3, 15 patients had a CSA pattern 4 (cfr web table 1 for pattern characteristics), 7 patients had a CSA pattern 5 (see web table 1).

At admission to the study, all the 50 AD patients and controls had instead an EEG CSA pattern 1, with stable alpha dominant frequency.

At follow-up, while none of DLB patients had an EEG CSA pattern 1, 21 AD patients showed an abnormal EEG pattern: 11 patients had a CSA 2, 5 had a CSA pattern 3, 4 a CSA pattern 4, 1 a CSA pattern 5, confirming the strongest statistical differences in the comparison between dementia subgroups, as concerned to EEG characteristics, are found at onset of dementia.

All the control subjects showed a normal EEG pattern (CSA pattern 1) at follow-up.

EEG recordings (at both admission and at follow-up) in the 5 patients who developed a neurodegenerative dementia different than DLB or AD showed an EEG pattern 1 with stable alpha activity.

Neuropsychological profile of the 5 MCI subjects who developed other types of dementia.

|  |  | Mean | Std Deviation |
| --- | --- | --- | --- |
| Age |  | 71.2 | 5.3 |
| Education |  | 7.8 | 3.3 |
| MMSE | Admission | 27.4 | 2.4 |
|  | Follow-up | 16.0 | 5.3 |
| CAF | Admission | 0.0 | 0.0 |
|  | Follow-up | 0.00 | 0.0 |
| UPDRS | Admission | 13.6 | 4.3 |
|  | Follow-up | 21.4 | 4.6 |
| FAB | Admission | 10.2 | 4.0 |
|  | Follow-up | 7.4 | 4.3 |
| GDS | Admission | 2.4 | 0.5 |
|  | Follow-up | 1.6 | 0.9 |
| geriatric depression scale | Admission | 1.2 | 0.8 |
|  | Follow-up | 0.2 | 0.4 |
| DRS-2 total score | Admission | 133.8 | 5.9 |
|  | Follow-up | 108.0 | 21.9 |
| DRS2 attention | Admission | 35.2 | 1.6 |
|  | Follow-up | 31.6 | 5.9 |
| DRS-2 initiation perseveration | Admission | 35.6 | 0.5 |
|  | Follow-up | 23.8 | 10.8 |
| DRS-2 Construction | Admission | 5.6 | 0.5 |
|  | Follow-up | 4.8 | 1.3 |
| DRS-2 Conceptualization | Admission | 38.0 | 0.7 |
|  | Follow-up | 31.2 | 5.2 |
| DRS-2 Memory | Admission | 19.4 | 3.8 |
|  | Follow-up | 16.6 | 4.3 |

MMSE= Mini Mental State Examination; DRS-2= Dementia Rating Scale-2; FAB= Frontal Assessment Battery; GDS= Global Deterioration Scale ; UPDRS= Unified Parkinson’s Disease Rating Scale; CAF= Clinician Assessment of Fluctuations.

EEG CSA pattern in the 5 patients who developed other types of dementia.

|  |  | Mean | Std. Deviation |
| --- | --- | --- | --- |
| EEG CSA pattern | Admission | 1 |  |
|  | Follow-up | 1 |  |
|  |  |  |  |
| DF | admission | 9.2 | 0.84 |
|  | Follow-up | 9.2 | 0.84 |
|  |  |  |  |
| DFV | admission | 0.0 | 0.0 |
|  | Follow-up | 0.0 | 0.0 |

CSA =compressed spectral array; DF=Dominant Frequency; DFV= Dominant frequency variability.

Frequency table reporting the occurrence of clinical and laboratory features of DLB in the 5 MCI subjects developing other dementia types.

| **SPECT-DAT scan** | | | | | |
| --- | --- | --- | --- | --- | --- |
|  | | Frequency | Percent | Valid Percent | Cumulative Percent |
|  | Negative | 3 | 60.0 | 60.0 | 60.0 |
|  | positive | 2 | 40.0 | 40.0 | 100.0 |
|  | Total | 5 | 100.0 | 100.0 |  |
|  |  |  |  |  |  |
| **RBD_admission** | | | | | |
|  | | Frequency | Percent | Valid Percent | Cumulative Percent |
| Valid | negativa | 4 | 80.0 | 80.0 | 80.0 |
|  | positiva | 1 | 20.0 | 20.0 | 100.0 |
|  | Total | 5 | 100.0 | 100.0 |  |
|  |  |  |  |  |  |
| **VH_admission** | | | | | |
|  | | Frequency | Percent | Valid Percent | Cumulative Percent |
| Valid | negativa | 3 | 60.0 | 60.0 | 60.0 |
|  | positiva | 2 | 40.0 | 40.0 | 100.0 |
|  | Total | 5 | 100.0 | 100.0 |  |
|  |  |  |  |  |  |
| **RBD_follow-up** | | | | | |
|  | | Frequency | Percent | Valid Percent | Cumulative Percent |
| Valid | negativo | 4 | 80.0 | 80.0 | 80.0 |
|  | positivo | 1 | 20.0 | 20.0 | 100.0 |
|  | Total | 5 | 100.0 | 100.0 |  |
|  |  |  |  |  |  |
| **VH_follow-up** | | | | | |
|  | | Frequency | Percent | Valid Percent | Cumulative Percent |
| Valid | negativo | 3 | 60.0 | 60.0 | 60.0 |
|  | positivo | 2 | 40.0 | 40.0 | 100.0 |
|  | Total | 5 | 100.0 | 100.0 |  |
|  |  |  |  |  |  |

VH= Visual Hallucinations= SPECT-DAT scan= Single Photon Emission Computer Tomography-Dopamine Transporter; RBD= REM sleep Behaviour Disorder.
