# Supplementary material for: Quantitative electroencephalogram utility in predicting conversion of mild cognitive impairment to dementia with Lewy bodies
Source: Neurobiol Aging. 2015 Jan;36(1):434–45. doi: 10.1016/j.neurobiolaging.2014.07.009 (PMC4270449; doi:10.1016/j.neurobiolaging.2014.07.009)
Supplement: Web table 1 [file mmc5.doc]

Web table 1

**p-value**

**MCI-DLB**

**MCI-AD**

**MCI-NC**

**DLB**

**AD**

**Controls**

CAF

--

--

--

0.0001

--

--

UPDRS

--

0.0001

--

--

0.0001

--

MMSE

--

--

0.03

0.0001

0.0001

0.0001

DRS-2

--

--

--

0.0001

0.0001

0.0001

FAB

--

--

0.005

0.0001

0.0001

0.0001

GDS

--

--

0.01

0.0001

0.0001

0.0001

CAF

--

--

--

0.0001

--

--

UPDRS

0.0001

--

--

0.0001

--

--

MMSE

--

--

0.01

0.0001

0.0001

0.0001

DRS-2

--

--

--

0.0001

0.0001

0.0001

FAB

--

--

0.01

0.0001

0.0001

0.0001

GDS

--

--

0.0001

0.0001

0.0001

0.0001

CAF

--

--

--

0.0001

--

--

UPDRS

--

--

--

--

0.04

0.05

MMSE

0.03

0.01

--

0.0001

0.0001

--

DRS-2

--

--

--

0.0001

0.0001

--

FAB

0.005

0.01

--

--

--

0.001

GDS

0.01

0.0001

--

0.0001

0.0001

0.0001

CAF

0.0001

0.0001

0.0001

--

0.0001

0.0001

UPDRS

--

0.0001

--

--

0.0001

0.0001

MMSE

0.0001

0.0001

0.0001

--

--

0.0001

DRS-2

0.0001

0.0001

0.0001

--

--

0.0001

FAB

0.0001

0.0001

--

--

0.0001

GDS

0.0001

0.0001

0.0001

--

--

0.0001

CAF

--

--

--

0.0001

--

--

UPDRS

0.0001

--

0.04

0.0001

--

--

MMSE

0.0001

0.0001

0.0001

--

--

0.0001

DRS-2

0.0001

0.0001

0.0001

--

--

0.0001

FAB

0.0001

0.0001

--

--

--

0.0001

GDS

0.0001

0.0001

0.0001

--

--

0.0001

CAF

--

--

--

0.0001

--

--

UPDRS

0.0001

--

0.05

0.0001

--

--

MMSE

0.0001

0.0001

--

0.0001

0.0001

--

DRS-2

0.0001

0.0001

--

0.0001

0.0001

--

FAB

0.0001

0.0001

0.001

0.0001

0.0001

--

GDS

0.0001

0.0001

0.0001

0.0001

0.0001

--

**MCI-DLB**

**DLB**

**AD**

**Controls**

**MCI-AD**

**MCI-NC**

MCI = Mild Cognitive Impairment; MCI-NC= MCI non converters; MCI-DLB= MCI subjects who converted to DLB; MCI-AD= MCI subjects who converted to AD; DLB= Dementia with Lewy Bodies; AD= Alzheimer’s Disease; CAF= Clinician Assessment of Fluctuations; UPDRS= Unified Parkinson’s Disease Rating Scale; MMSE= Mini Mental State Examination; DRS-2= Dementia Rating Scale-2; FAB= Frontal Assessment Battery; GDS= Global Deterioration Scale.
